# p53 modulates kinase inhibitor resistance and lineage plasticity in NF1-related MPNSTs

**DOI:** 10.1038/s41388-024-03000-9

**Published:** 2024-03-13

**Authors:** Jamie L. Grit, Lauren E. McGee, Elizabeth A. Tovar, Curt J. Essenburg, Emily Wolfrum, Ian Beddows, Kaitlin Williams, Rachael T. C. Sheridan, Joshua L. Schipper, Marie Adams, Menusha Arumugam, Thomas Vander Woude, Sharavana Gurunathan, Jeffrey M. Field, Julia Wulfkuhle, Emanuel F. Petricoin, Carrie R. Graveel, Matthew R. Steensma

**Affiliations:** 1 Department of Cell Biology, Van Andel Research Institute, Grand Rapids, MI 49503 USA; 2 Bioinformatics & Biostatistics Core, Van Andel Research Institute, Grand Rapids, MI 49503 USA; 3 Flow Cytometry Core, Van Andel Research Institute, Grand Rapids, MI 49503 USA; 4 Genomics Core, Van Andel Research Institute, Grand Rapids, MI 49503 USA; 5 Department of Pharmacology, University of Pennsylvania Perelman School of Medicine, Philadelphia, PA 19104 USA; 6 Center for Applied Proteomics and Molecular Medicine, George Mason University, Manassas, VA 20110 USA; 7 Helen DeVos Children’s Hospital, Corewell Health System, Grand Rapids, MI 49503 USA; 8 Michigan State University College of Human Medicine, Grand Rapids, MI 49503 USA

**Keywords:** Sarcoma, Oncogenes, Growth factor signalling, Differentiation, Genetic markers

## Abstract

Malignant peripheral nerve sheath tumors (MPNSTs) are chemotherapy resistant sarcomas that are a leading cause of death in neurofibromatosis type 1 (NF1). Although NF1-related MPNSTs derive from neural crest cell origin, they also exhibit intratumoral heterogeneity. *TP53* mutations are associated with significantly decreased survival in MPNSTs, however the mechanisms underlying *TP53-*mediated therapy responses are unclear in the context of *NF1*-deficiency. We evaluated the role of two commonly altered genes, *MET* and *TP53*, in kinome reprograming and cellular differentiation in preclinical MPNST mouse models. We previously showed that *MET* amplification occurs early in human MPNST progression and that *Trp53* loss abrogated MET-addiction resulting in MET inhibitor resistance. Here we demonstrate a novel mechanism of therapy resistance whereby p53 alters MET stability, localization, and downstream signaling leading to kinome reprogramming and lineage plasticity. *Trp53* loss also resulted in a shift from RAS/ERK to AKT signaling and enhanced sensitivity to MEK and mTOR inhibition. In response to MET, MEK and mTOR inhibition, we observed broad and heterogeneous activation of key differentiation genes in *Trp53*-deficient lines suggesting *Trp53* loss also impacts lineage plasticity in MPNSTs. These results demonstrate the mechanisms by which p53 loss alters MET dependency and therapy resistance in MPNSTS through kinome reprogramming and phenotypic flexibility.

## Introduction

Malignant peripheral nerve sheath tumors (MPNSTs) are aggressive, chemoresistant sarcomas arising from Schwann cells that are the leading cause of death in patients with Neurofibromatosis Type 1 (NF1) [1]. NF1 is an autosomal dominant tumor predisposition syndrome caused by inactivating mutations in the *NF1* gene [2–4]. *NF1* is a tumor suppressor gene that encodes neurofibromin, a critical negative regulator of RAS [5]. NF1-related MPNSTs exhibit deregulated RAS signaling caused by loss of heterozygosity of *NF1* along with additional tumor suppressor loss (*TP53, CDKN2A, SUZ12, PTEN*) and receptor tyrosine kinase (RTK) amplification (*MET, EGFR, PDGFR*) [6–12]. As such, targeted therapies against RTKs and RAS effectors including MEK have been proposed as a treatment option for MPNSTs. Even with promising preclinical results, clinical trials featuring tyrosine kinase inhibitors have not been successful to date [13–16]. Because of their aggressive clinical behavior, the 5 year survival rate remains only at 10–50% [17–20].

Although histologic and genomic MPNST subtypes have been described, these categories are not therapeutically relevant. The MPNST chemotherapy regimen has remained largely unchanged since the incorporation of doxorubicin in the 1980’s. The lack of actionable MPNST subtypes remains a major barrier to effective treatment, particularly given the vast differences in kinase signaling between histologically identical tumors [14, 21–25]. Additionally, MPNSTs are known to exhibit divergent states of differentiation leading to intratumoral heterogeneity. For example, MPNSTs can contain cellular regions comprised of malignant muscle, bone, fat, nerve and cartilage cells [26]. It has long been suspected that the differentiation states of various MPNST histologic subtypes contribute to therapy resistance, but confirmatory data is lacking. Thus the identification of predictive biomarkers for MPNSTs continues to be an area of intense study [27]. 25–60% of MPNSTs are p53-deficient [9, 10, 28], which is associated with significantly diminished survival [28–30] and poor response to neo-adjuvant chemotherapy [31]. Additionally, MET [32] and PI3K/AKT/mTOR pathway [33] activation are both associated with poor prognosis, and animal modeling indicates that genetic activation of these pathways in the context of *NF1* loss drives MPNST growth and is targetable therapeutically [34, 35]. *MET* or its ligand, *HGF*, are amplified in 25–50% of MPNSTs [7, 8], which results in activation of downstream effectors including RAS, PI3K, and STAT3. PI3K may also be activated in MPNST via loss of *PTEN*, its major upstream negative regulator [36], or via amplification of other RTKs including *EGFR* [37]. Collectively, the PI3K/AKT/mTOR pathway is activated in 50% of MPNSTs [33]. Crosstalk between p53 and PI3K/AKT/mTOR occurs in a cell type and stress-dependent manner, in which positive or negative regulation of either pathway is highly contextual and may be reciprocal [38]. Defining the role of these pathways in therapeutic response is critical to predicting effective targeted therapies and predictive biomarkers for future MPNST trials.

Consistent with these clinical observations, we previously found that a *Trp53*-deficient mouse model of NF1-related MPNST exhibited faster tumor growth and was more resistant to chemotherapy compared to *Trp53*-intact models. Interestingly, the *Trp53*-deficient model was also less sensitive to single agent and combination MEK and MET inhibition despite sustained repression of ERK phosphorylation. *Trp53*-deficient tumors also exhibited unusually high AKT activation both at baseline and in response to targeted therapy [25, 34]. In this study, we aimed to define the role of p53 in regulating kinase signaling, targeted therapy response, and cellular differentiation in NF1-related MPNSTs. Transcriptomic and phospho-proteomic analysis revealed multiple mechanisms of resistance, including deregulation of MET stability and localization, deregulated PI3K/AKT/mTOR signaling, and altered lineage plasticity. In contrast to these results, we found that p53 loss actually increased sensitivity to mTOR inhibition, which was associated with broad and persistent kinome activation. Excitingly, combined mTOR and MEK inhibition reversed clonal selection for p53-deficiency and was the most effective drug combination in all models, regardless of p53 status. Lineage plasticity, as defined by transcriptional profiling, was closely linked to tyrosine kinase inhibitor resistance. Collectively, these data suggest that p53 acts as a master regulator of tyrosine kinase signaling and mediates oncogene-addiction and cell fate in MPNSTs.

## Results

### p53-deficiency is associated with MET inhibitor resistance in MPNSTs

**Fig. 1:**
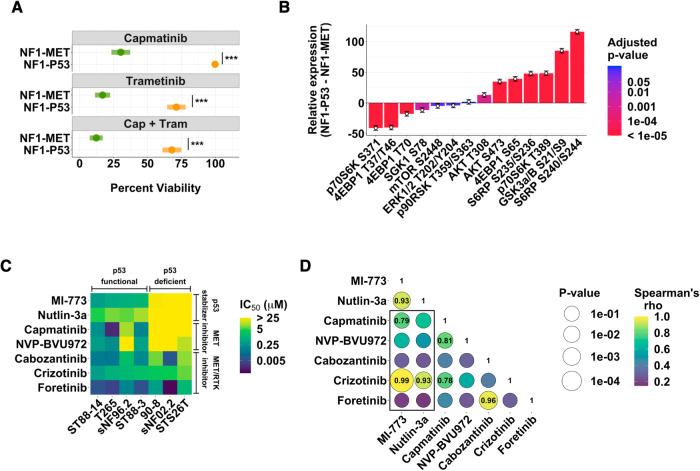
p53 deficiency is associated with MET inhibitor resistance in MPNSTs. **A** Percent viability of NF1-MET and NF1-P53 cells after 72 h of capmatinib (100 nM), trametinib (40 nM) or combination (capmatinib 100 nM, trametinib 40 nM) treatment. **B** Change in phospho-site activation of MET effectors in NF1-P53 cells relative to NF1-MET cells upon capmatinib (100 nM), trametinib (100 nM), or combination (capmatinib 100 nM, trametinib 100 nM) treatment for 2 and 48 h. See also Supplementary Fig. 2. **C** IC_50_ of p53 stabilizing drugs and MET inhibitors against a panel of human MPNST cell lines. **D** Spearman’s correlations (color) and significance (size) between the IC_50_ of the drugs in (**C**). The rho value of correlations with a p-value < 0.5 are indicated in their respective bubble. The black box indicates the correlations between p53 stabilizing drugs and MET inhibitors. ***p < 0.001.

Previously, we compared response to MET and MEK inhibition in tumor xenografts derived from MPNSTs of genetically engineered mouse models of NF1, including a *Met*-amplified, *Trp53*-wildtype model (NF1-MET; genotype: *Nf1*^*fl/ko*^*;lox-stop-loxMET*^*tg/+*^*;Plp-creERT*^*tg/+*^) and a *Trp53* deficient model (NF1-P53; genotype: *Nf1*^*ko/+*^*;p53*^*R172H*^*;Plp-creERT*^*tg/+*^). NF1-P53 MPNSTs were less sensitive to both MET and MEK inhibition in vivo [34]. To further investigate the molecular mechanisms of drug resistance, we generated tumor cell line isolates from these murine MPNSTs. Cell viability analysis confirmed that the MPNST cell lines maintained their drug sensitivity phenotypes in vitro, as the NF1-P53 cell line was resistant to single agent MET (capmatinib) and MEK (trametinib) inhibition as well as combination therapy (Fig. 1A). A targeted analysis of the RAS/ERK and PI3K/AKT pathways with reverse phase protein arrays (RPPA) was used to identify both immediate (2 h) and adaptive (48 h) kinome responses to MET and MEK inhibition. After controlling for drug treatment and exposure time, several PI3K effectors (including AKT and S6) were significantly activated in the NF1-P53 cells, suggesting PI3K/AKT pathway activation may promote resistance to MET and MEK inhibition (Fig. 1B, Supplementary Fig. 2).

To determine if p53 also regulates sensitivity to MET inhibition in human MPNSTs, we screened a panel of 6 NF1-related and 1 sporadic (STS26T) MPNST cell lines for functional p53 based on sensitivity to the p53 stabilizing drugs, MI-773 and nutlin-3a. These p53 stabilizers inhibit MDM2-mediated p53 degradation and are selectively active in p53 wild-type cells [39]. Based on the IC_50_ of these drugs, we classified four cell lines as being “p53-functional” and three cell lines as being “p53-deficient” (Fig. 1C, Supplementary Fig. 3A), which was consistent with a recent genomic analysis of MPNST cell lines [40]. In addition, we screened the cell lines for sensitivity to of a panel of MET inhibitors. All but one of the p53-intact cell lines were sensitive to MET inhibition while, the p53-deficient cells were profoundly resistant to MET inhibition (Fig. 1C, Supplementary Fig. 3A). The IC_50_ between several of the p53 stabilizers and MET inhibitors significantly correlated, similar to drugs within the same class (Fig. 1D, Supplementary Fig. 3B), suggesting a critical role for p53 in regulating MET-dependency.

### p53 loss drives MET inhibitor resistance in MPNSTs

**Fig. 2:**
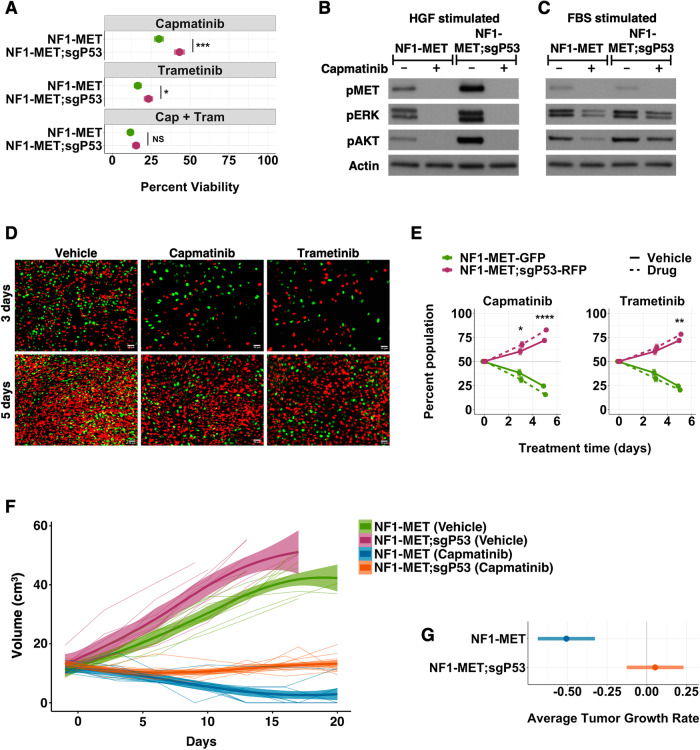
p53 loss drives MET inhibitor resistance in MPNSTs. **A** Percent viability of NF1-MET and NF1-MET;sgP53 cells after 72 h of capmatinib (100 nM), trametinib (40 nM) or combination (capmatinib 100 nM, trametinib 40 nM) treatment. **B**, **C** Western blot of NF1-MET and NF1-MET;sgP53 cells treated with capmatinib (100 nM) for 2 h and stimulated with HGF (**B**) or 10% FBS (**C**) for 15 min. Images (**D**) and flow cytometry analysis (**E**) of GFP labeled NF1-MET and RFP labeled NF1-MET;sgP53 cells after 3 and 5 days of treatment with vehicle (DMSO), capmatinib (100 nM), or trametinib (40 nM). **F** Individual tumor growth curves, LOESS curves, and 95% ribbons for vehicle or capmatinib (30 mg/kg BID) treated NF1-MET and NF1-MET;sgP53 xenografts. **G** Pairwise comparison of growth trend estimates in capmatinib treated NF1-MET (*p*-value = 0.0083) and NF1-MET;sgP53 (*p*-value = 0.089) tumors to 0 (grey line). *p < 0.05, **p < 0.01, ***p < 0.001, ****p < 0.0001.

To evaluate the impact of p53 loss-of-function in the context of MET-addiction, we used CRISPR-Cas9-mediated knockout of p53 in murine NF1-MET cells to create the NF1-MET;sgP53 line. p53 protein levels as well as the p53 target gene, p21, were diminished in NF1-MET;sgP53 cells (Supplementary Fig. 4A, B). Cell viability analysis demonstrated that NF1-MET;sgP53 cells were significantly less sensitive to MET inhibition than NF1-MET cells; however, combined MET-MEK inhibition restored drug sensitivity (Fig. 2A). Interestingly, the magnitude of resistance was less in the isogenic cells compared to the NF1-MET and NF1-P53 cells (Supplemental Fig. 4C), suggesting the possibility of additional genetic modifiers in the NF1-P53 cell line. *CDKN2A* loss is a key step in pre-MPNST transformation [41] and contributes to drug resistance in other tumor types [42, 43]. We evaluated the NF1-MET and NF1-P53 cell lines for spontaneous *Cdkn2a* loss, however *Cdkn2a* was deleted only in the NF1-MET cell line (Supplemental Fig. 4D), indicating *Cdkn2a* loss does not contribute to increased drug resistance in the NF1-P53 cell line. We next evaluated the impact of p53 on HGF-induced MET signaling and observed augmented MET signaling in NF1-MET;sgP53 cells, yet capmatinib inhibited HGF-dependent ERK and AKT activation regardless of p53 status (Fig. 2B). Stimulation with FBS (which contains other growth factors as well as HGF) also induced pERK and pAKT. Interestingly, the effect of capmatinib was quite modest in NF1-MET;sgP53 cells, suggesting capmatinib resistance may be partially mediated by parallel pathway activation that converges on ERK and AKT (Fig. 2C).

Resistant clonal populations within heterogeneous MPNSTs may explain the clinical failures of targeted kinase inhibitors in MPNSTs [44]. To determine if p53 is a key driver of clonal selection and drug resistance in MPNSTs, we performed a clonal competition assay using labeled isogenic NF1-MET-GFP and NF1-MET;sgP53-RFP cell lines. Notably, when cultured separately, NF1-MET;sgP53 cells do not have a significant proliferative advantage compared to NF1-MET cells (Supplementary Fig. 4E). In contrast, when cocultured NF1-MET;sgP53-RFP cells had a strong growth advantage, which was significantly enhanced by both MET or MEK inhibition (Fig. 2D, E). After 5 days of capmatinib treatment, NF1-MET;sgP53-RFP cells comprised 83% of the culture (Fig. 2D, E), indicating that p53 loss drives strong clonal selection with MET inhibition.

To evaluate p53 loss and capmatinib sensitivity in vivo, we treated orthotopic MPNST xenografts with capmatinib. Tumor growth rate was significantly increased in the NF1-MET;sgP53 tumors compared to the parental tumors (*p*-value = 4.7e-4) (Fig. 2F; Supplementary Fig. 4F, G). Capmatinib strongly inhibited tumor growth in both the NF1-MET and NF1-MET;sgP53 models (Fig. 2F; Supplementary Fig. 4F), however only the NF1-MET tumors regressed on treatment (*p*-value = 0.0083), while the NF-MET;sgP53 tumors remained stable throughout treatment, with a subset of tumors actually showing increased growth (Fig. 2F, G; Supplementary Fig. 4F). These data confirm our in vitro findings and further demonstrate that p53 loss promotes MET inhibitor resistance.

### Met inhibition induces p53-dependent lineage plasticity

**Fig. 3:**
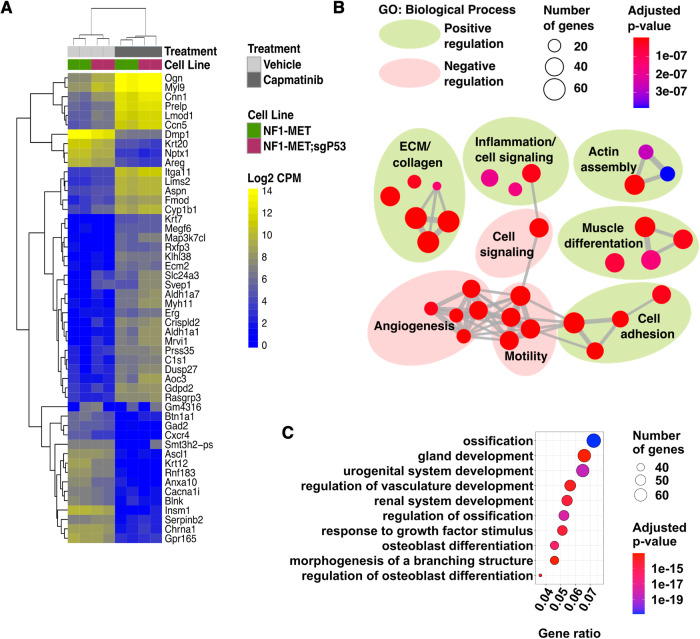
Met inhibition induces p53-dependent linage plasticity. **A** Unsupervised hierarchical clustering of top 50 differentially expressed genes in NF1-MET and NF1-MET;sgP53 cells treated with capmatinib (100 nM) for 24 h. **B** GO term enrichment analysis of capmatinib-induced genes. The top 30 most significantly enriched biological process terms (by adjusted *p*-value) are shown. Connecting grey lines represent relatedness of the pathways, while dot size indicates the number of genes differentially expressed in the pathway. See Supplementary Fig. 5B for individual terms. **C** Top 10 most significantly enriched biological process GO terms derived from genes that were decreased in capmatinib treated NF1-MET;sgP53 cells. See Supplementary Fig. 5C for expanded top 30 terms.

To understand how loss of p53 drives resistance we used RNA-seq to examine capmatinib-induced transcriptional changes in NF1-MET and NF1-MET;sgP53 cells. Unsupervised hierarchical clustering identified strong clustering by treatment followed by genotype, with p53 status modifying expression of some gene subsets (Fig. 3A). GO term enrichment analysis of genes upregulated by MET inhibition identified biological processes related to positive regulation of actin organization, cell adhesion, collagen deposition/ossification, and muscle differentiation (Fig. 3B, Supplementary Fig. 5A). We next examined genes that were downregulated in capmatinib treated NF1-MET;sgP53 cells and identified biological processes related to bone and kidney development (Fig. 3C, Supplementary Fig. 5B). Together, these data suggest that response to MET inhibition may be partially mediated through the induction of linage plasticity pathways in MPNSTs and that p53 loss disrupts this process to promote drug resistance. As differentiation and cell cycle arrest are coupled, we next examined whether p53 loss altered expression of known p53 target genes that promote cell cycle arrest. Indeed, expression of the cell cycle regulators *Cdkn1a* and *Zmat3* were lost in the NF1-MET;sgP53 cells, while expression of genes involved in senescence or apoptosis were unchanged (Supplementary Fig. 5C).

### p53 regulates MET stability and localization

**Fig. 4:**
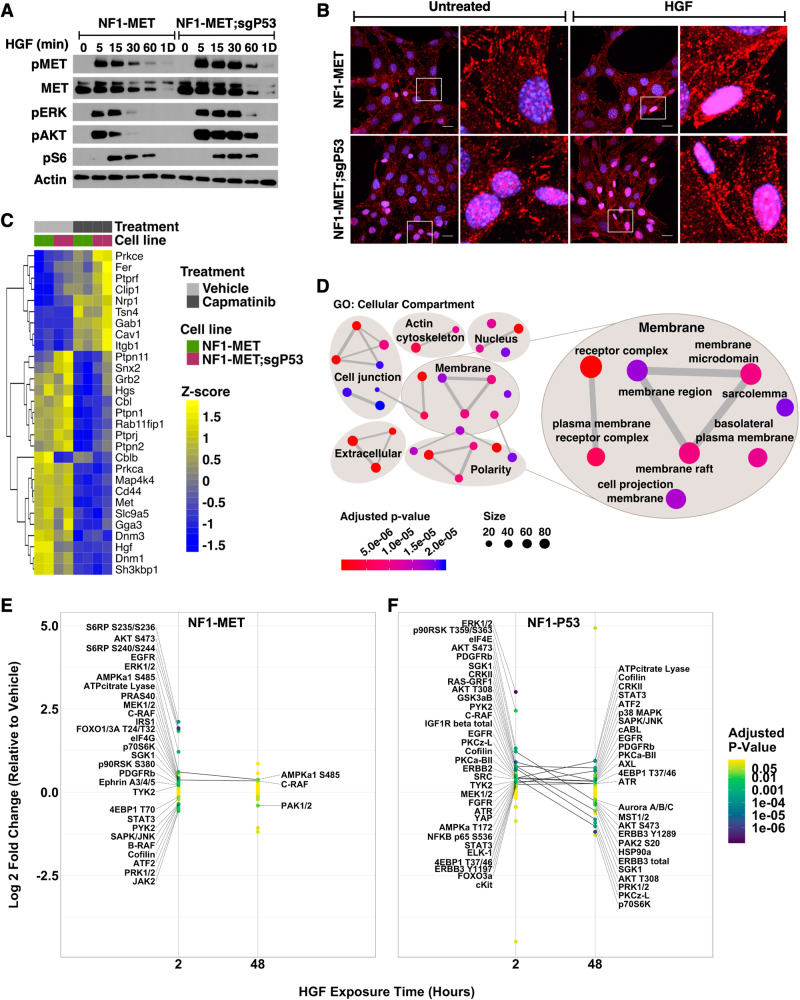
p53 regulates MET stability, localization, and effector signaling. **A** Time course western blot of NF1-MET and NF1-MET;sgP53 cells stimulated with HGF for 5 min to 1 day. **B** Representative images of phospho-MET localization (red) after 5 min of HGF stimulation. **C** Expression of genes associated with MET stability and localization after 24 h of vehicle (DMSO) or capmatinib (100 nM) treatment. **D** GO analysis of Cellular Compartment terms downregulated in the NF1-MET;sgP53 cell line compared to the parental NF1-MET line. See Supplementary Fig. 5D for individual terms. Change in phospho-protein expression in NF1-MET (**E**) and NF1-P53 (**F**) cells after HGF treatment. Significantly increased or decreased proteins at each time point are labeled and proteins that are significant at both the 2 and 48 h time points are connected. See Table 1 for phosphosites. Color indicates P-value.

RTK expression, activation, and recycling are tightly regulated processes that ensure RTK regulation in normal physiological conditions. Deregulation of this cycle of RTK activation and recycling is often observed in cancers, such as the MET exon 14 deletions found in lung cancers [45]. To determine whether RTK spaciotemporal regulation promoted the enhanced MET signaling observed in p53-deficient MPNST cells (Figs. 1B & 2B, C), we measured the kinetics of MET activation and turnover. In NF1-MET cells an immediate, and expected, increase in MET activation was observed within 5 min of HGF treatment that quickly diminished along with total MET levels over time (Fig. 4A). In contrast, HGF-treatment induced a drastic pMET increase that remained elevated for 60 min in NF1-MET;sgP53 cells. Both phosphorylated and total MET were persistently elevated in the NF1-MET;sgP53 cells, which corresponded to increased and prolonged activation of both ERK and AKT (Fig. 4A). Activation of the mTOR effector S6 was similar between the two cell lines.

To examine whether p53 loss also alters MET subcellular localization, we performed immunostaining of MET after HGF-treatment. In normal conditions, HGF treatment results in MET localization and activation at the plasma membrane; however MET can also be internalized to the nucleus, where it’s function is incompletely understood [46]. At baseline, pMET was localized to the cytoplasm in the NF1-MET cells, whereas we observed both nuclear and cytoplasmic staining in NF1-MET;sgP53 cells. Interestingly, HGF dramatically increased nuclear MET localization in the p53-deficient NF1-MET;sgP53 cells, while treatment induced nuclear MET localization only in a small percentage of NF1-MET cells (Fig. 4B). In several cancers, nuclear MET is associated with drug resistance and poor prognosis [47–51]. These results suggest that p53 loss induces nuclear MET localization, promoting tumor aggressive phenotypes in MPNST cells.

To understand how loss of p53 promotes increased stability and nuclear localization of MET, we used RNA-seq to examine the expression of genes involved in MET activation and turnover. Capmatinib treatment induced sweeping compensatory expression changes in both cell lines (Fig. 4C). In NF1-MET;sgP53 cells, expression of genes critical for MET degradation, *Sh3kpb1* and *Cblb*, [52–54] were significantly downregulated compared to the parental cell line. Conversely, *Prkce* expression, which is required for nuclear MET translocation [55], was significantly upregulated in the NF1-MET;sgP53 cells in the presence of capmatinib. GO cellular compartment enrichment analysis revealed that p53 loss promoted significant downregulation in plasma membrane and receptor organization pathways (Fig. 4D, Supplementary Fig. 5D).

MET and the RTK EGFR share much of the same recycling machinery [54, 56], and *EGFR* is also frequently amplified in MPNST [37]. To determine if p53 loss also enhanced EGFR activation, we treated the NF1-MET and NF1-MET;sgP53 cells with EGF. Strikingly, EGF induced much stronger EGFR phosphorylation in the NF1-MET;sgP53 cells than the parental cell line (Supplementary Fig. 6), similar to the increased MET activation upon HGF treatment. This corresponded with increased and prolonged activation of pERK, pAKT, and pS6. These results suggest an important role for p53 in regulating both MET and EGFR signaling in MPNSTs and may partially explain resistance to EGFR inhibition in MPNSTs [57].

To more broadly examine the effect of p53 expression on MET signaling, we used RPPA to evaluate the response of 98 protein phosphosites to short and extended HGF stimulation. After 2 h of HGF treatment, numerous phosphosites were significantly upregulated in both the NF1-MET and NF1-P53 cell lines, yet the number of upregulated sites as well as the magnitude of change was higher in the p53-deficient cells (Fig. 4E, F, Table 1). Additionally, phosphorylation of several proteins, including STAT3, JAK2, and B-RAF, were significantly decreased in the NF1-MET cell line after just 2 h HGF treatment, (Fig. 4E, Table 1) suggesting that rapid negative feedback signaling in response to MET activation is p53-dependent. Remarkably, after 48 h of HGF treatment, only 3/98 phospho-sites were significantly changed in the NF1-MET cells compared to 27/98 sites in the NF1-P53 cells (Fig. 4E, F, Table 1). Notably, STAT3 Y705 phosphorylation, which is sustained by perinuclear MET [58], was among the most significantly increased phosphosites in the p53-deficient cells after 48 h of HGF exposure (Fig. 4F, Table 1). Collectively, these data suggest that p53 loss moderates MET addiction by modulating the location, timing, and magnitude of MET effector signaling.

**Table 1:** Reverse phase protein array fold change in expression relative to vehicle.

Cell.line	Time	gene	logFC	t	SE	adj.P.Val
NF1-MET	2	AKT S473	1.922302123	13.17752484	0.145877329	8.98E-07
NF1-MET	2	S6RP S240/S244	1.901331348	12.18886476	0.155989207	1.13E-06
NF1-MET	2	S6RP S235/S236	2.113685262	7.427556054	0.284573451	0.000191344
NF1-MET	2	SAPK/JNK T183/Y185	−0.357780978	−7.176927867	0.04985155	0.00020508
NF1-MET	2	PRAS40 T246	0.427694519	6.446338782	0.066346888	0.000486589
NF1-MET	2	ERK1/2 T202/Y204	1.211520394	6.189667411	0.195732713	0.0006043
NF1-MET	2	p90RSK S380	0.279372169	5.196687333	0.053759665	0.002642982
NF1-MET	2	IRS1 S612	0.351537864	5.113142028	0.068751829	0.002654418
NF1-MET	2	Cofilin S3	−0.386870886	−5.04811324	0.076636729	0.002654418
NF1-MET	2	B-RAF S445	−0.373076735	−4.73781656	0.078744445	0.004054089
NF1-MET	2	FOXO1 T24/FOXO3a T32	0.350437141	4.692337306	0.074682854	0.004054089
NF1-MET	2	p70S6K S371	0.34623766	4.503365928	0.076884194	0.00497755
NF1-MET	2	TYK2 Y1054/Y1055	0.207025297	4.48444698	0.046165179	0.00497755
NF1-MET	2	EGFR Y1068	1.833693254	4.169044594	0.439835366	0.008210613
NF1-MET	2	AMPKa1 S485	0.60751555	4.096781071	0.148290948	0.008754974
NF1-MET	2	MEK1/2 S217/S221	0.382514877	4.021501455	0.095117428	0.009434676
NF1-MET	2	JAK2 Y1007	−0.563544454	−3.981228378	0.141550396	0.009459995
NF1-MET	2	ATF2 T71	−0.493146086	−3.956642856	0.124637503	0.009459995
NF1-MET	2	C-RAF S338	0.366910981	3.629966153	0.101078348	0.016447315
NF1-MET	2	elF4G S1108	0.347419148	3.590275085	0.096766721	0.016447315
NF1-MET	2	PRK1 T774/PRK2 T816	−0.554399931	−3.579608311	0.154877261	0.016447315
NF1-MET	2	SGK1 S78	0.328678952	3.158633033	0.10405734	0.033940163
NF1-MET	2	Ephrin A3 Y799/A4 Y799/A5 Y833	0.211585037	3.150067002	0.067168424	0.033940163
NF1-MET	2	4EBP1 T70	−0.141851826	−3.118418469	0.045488387	0.03454584
NF1-MET	2	PDGFRb Y751	0.224593878	3.041081528	0.073853291	0.038423831
NF1-MET	2	PYK2 Y402	−0.357038387	−2.963657163	0.12047223	0.042611794
NF1-MET	2	ATPcitrate Lyase S454	0.578983064	2.92971822	0.197624147	0.042611794
NF1-MET	2	PKCa-BII T638/T641	−0.268588053	−2.92711569	0.091758605	0.042611794
NF1-MET	2	STAT3 Y705	−0.199537985	−2.901091404	0.068780316	0.043228251
NF1-MET	2	p90RSK T359/S363	0.299326773	2.790135479	0.107280372	0.051579862
NF1-MET	2	NFKB p65 S536	0.384217692	2.734680663	0.140498193	0.055441102
NF1-MET	2	AKT T308	−0.140812972	−2.609527129	0.053961107	0.068011589
NF1-MET	2	Paxillin Y118	−0.425764858	−2.462668149	0.172887629	0.086838723
NF1-MET	2	Chk1 S345	−0.385540662	−2.430346807	0.158636068	0.089515615
NF1-MET	2	AXL Y702	−0.224327571	−2.361084704	0.095010387	0.098886672
NF1-MET	2	PAK1 S199/S204-PAK2 S192/S197	0.288540675	2.336184697	0.123509359	0.100219981
NF1-MET	2	cABL Y245	0.256465918	2.323768124	0.110366398	0.100219981
NF1-MET	2	FRS2a Y436	0.130796537	2.147269734	0.060912951	0.134777023
NF1-MET	2	FOXO1 S256	−0.159413387	−2.108642238	0.075600016	0.137625892
NF1-MET	2	Raf S259	−0.354879646	−2.107342191	0.168401529	0.137625892
NF1-MET	2	AMPKb1 S108	0.227215981	2.077323391	0.109379205	0.141727924
NF1-MET	2	Acetyl CoAC S79	0.194756604	1.987884072	0.097971812	0.162333371
NF1-MET	2	eNOS S1177	−0.132242743	−1.90489787	0.069422485	0.183577551
NF1-MET	2	elF4E S209	0.20749804	1.888237211	0.109889816	0.184719755
NF1-MET	2	LIMK1 T508/LIMK2 T505	0.502971608	1.874645387	0.268302267	0.184957224
NF1-MET	2	ELK-1 S383	−0.126221239	−1.848627836	0.06827834	0.189327013
NF1-MET	2	p70S6K T389	−0.223079564	−1.830005003	0.121901068	0.19138652
NF1-MET	2	CRKII Y221	0.161829588	1.71803078	0.094194813	0.224956683
NF1-MET	2	AMPKa T172	−0.106060366	−1.711454358	0.061970899	0.224956683
NF1-MET	2	STAT5 Y694	−0.12552217	−1.648615013	0.076137952	0.245083145
NF1-MET	2	STAT4 Y693	0.188068483	1.636372925	0.114930087	0.245246876
NF1-MET	2	PKCz-L T410/T403	−0.340169334	−1.613562251	0.210818847	0.249846724
NF1-MET	2	MST1 T183/MST2 T180	−0.116366722	−1.55288187	0.074935978	0.270957325
NF1-MET	2	HSP27 S82	0.204477521	1.433895702	0.142602786	0.314784611
NF1-MET	2	PAK2 S20	0.075958983	1.427312817	0.053218175	0.314784611
NF1-MET	2	PTEN S380	0.09618027	1.425906581	0.067452013	0.314784611
NF1-MET	2	Aurora AT288/BT232/CT198	−0.075020147	−1.388531212	0.05402842	0.328084638
NF1-MET	2	IGF1R beta total	−0.109648759	−1.313599187	0.083472005	0.358184767
NF1-MET	2	SUMO2/3 total	−0.088966648	−1.309940521	0.067916555	0.358184767
NF1-MET	2	PDK1 S241	0.119972047	1.283834566	0.093448214	0.36659027
NF1-MET	2	EGFR	−0.115202589	−1.212201788	0.095035819	0.395688162
NF1-MET	2	HSP90a T5/T7	0.061561236	1.211513227	0.050813507	0.395688162
NF1-MET	2	STAT1 Y701	0.091864833	1.197848256	0.076691544	0.397412797
NF1-MET	2	p38 MAPK T180/Y182	−0.090398979	−1.159134316	0.077988356	0.414215723
NF1-MET	2	MCSFR Y732	0.09161124	1.127143873	0.081277326	0.425867883
NF1-MET	2	FOXO3a S256	0.064443492	1.113218684	0.057889337	0.425867883
NF1-MET	2	ERBB2 Y1248	0.054571225	1.102954623	0.049477307	0.425867883
NF1-MET	2	EGFR Y1148	0.057698423	1.098267471	0.052535857	0.425867883
NF1-MET	2	MET Y1234/Y1235	0.062520874	1.084193355	0.057665797	0.428245587
NF1-MET	2	ALK Y1586	0.048094682	1.007265605	0.047747766	0.453860046
NF1-MET	2	IGF1R Y1135/Y1136-IR Y1150/Y1151	0.048104918	0.995876617	0.048304094	0.453860046
NF1-MET	2	PKCa-BII T638/T641.1	0.100953368	0.992699373	0.101695811	0.453860046
NF1-MET	2	ERBB3 total	−0.089390304	−0.991566511	0.090150588	0.453860046
NF1-MET	2	SEK1/MKK4 S80	0.062495857	0.984466402	0.06348196	0.453860046
NF1-MET	2	mTOR S2448	0.11995107	0.983356482	0.121981268	0.453860046
NF1-MET	2	ATR S428	0.06010448	0.916346836	0.065591409	0.48923586
NF1-MET	2	Insulin Rec beta total	0.139451477	0.907646742	0.153640696	0.48923586
NF1-MET	2	4EBP1 S65	−0.071161791	−0.898915627	0.079164039	0.48923586
NF1-MET	2	4EBP1 T37/46	0.086559049	0.837613545	0.103340077	0.517529735
NF1-MET	2	cKit Y719	−0.047101592	−0.822027741	0.057299273	0.517529735
NF1-MET	2	EGFR Y1173	−0.060353872	−0.821221992	0.073492761	0.517529735
NF1-MET	2	FAK Y576/Y577	−0.084928722	−0.817651773	0.103869061	0.517529735
NF1-MET	2	ERBB3 Y1197	0.03893665	0.745070258	0.052259031	0.5603899
NF1-MET	2	RAS-GRF1 S916	−0.037999327	−0.725068367	0.052407923	0.567581099
NF1-MET	2	ERBB3 Y1289	−0.047112954	−0.681180319	0.069163704	0.591686368
NF1-MET	2	ATF2 T69/71	0.287905702	0.553428151	0.520222365	0.678726405
NF1-MET	2	cKit Y719.1	−0.026938522	−0.523060932	0.05150169	0.694087297
NF1-MET	2	PTEN total	−0.038462342	−0.465095131	0.082697795	0.730972672
NF1-MET	2	JAK1 Y1022/Y1023	−0.02886501	−0.446166932	0.064695539	0.737499192
NF1-MET	2	ERK 1/2 total	−0.021646902	−0.430497215	0.050283489	0.741470992
NF1-MET	2	cAbl T735	0.015672364	0.28602473	0.054793736	0.840427895
NF1-MET	2	PAK1 T423/PAK2 T402	0.015374589	0.283991317	0.054137532	0.840427895
NF1-MET	2	IGF1R Y1131/IR Y1146	−0.018281078	−0.233796222	0.078192357	0.871708101
NF1-MET	2	YAP S127	0.020694077	0.215920833	0.095841039	0.876766942
NF1-MET	2	FGFR Y653/Y654	0.010820267	0.192137219	0.056315308	0.885142626
NF1-MET	2	GSK3aB S21/S9	−0.024481708	−0.182183964	0.134379047	0.885142626
NF1-MET	2	SRC Family Y416	−0.017649341	−0.160619595	0.109882865	0.892964663
NF1-MET	2	SRC Y527	0.003733285	0.060600902	0.061604442	0.962342908
NF1-MET	2	ERBB4 total	−0.001936876	−0.017160856	0.112865928	0.986575511
NF1-P53	2	ERK1/2 T202/Y204	3.010364583	17.13568196	0.175678131	5.99E-08
NF1-P53	2	PDGFRb Y751	0.904525529	10.11412579	0.089431904	1.28E-05
NF1-P53	2	GSK3aB S21/S9	0.669624449	8.432233252	0.079412467	6.15E-05
NF1-P53	2	AKT T308	0.71647495	6.663274863	0.107525949	0.000511218
NF1-P53	2	AKT S473	1.218304914	6.49272911	0.187641421	0.000526599
NF1-P53	2	elF4E S209	1.313683465	6.217691262	0.211281553	0.000665189
NF1-P53	2	IGF1R beta total	0.489705544	5.435102443	0.090100518	0.001972004
NF1-P53	2	C-RAF S338	0.494346801	5.203431476	0.095004	0.002497863
NF1-P53	2	MEK1/2 S217/S221	0.340689965	5.079591142	0.067070352	0.002497863
NF1-P53	2	FGFR Y653/Y654	0.334713668	5.078976972	0.065901789	0.002497863
NF1-P53	2	RAS-GRF1 S916	0.718165835	4.879138263	0.147191122	0.003191807
NF1-P53	2	CRKII Y221	0.780558668	4.761242432	0.163940123	0.003585727
NF1-P53	2	TYK2 Y1054/Y1055	0.343089769	4.350194218	0.07886769	0.006821601
NF1-P53	2	p90RSK T359/S363	2.446636721	4.232685332	0.578034163	0.007818765
NF1-P53	2	YAP S127	0.30150881	4.078700928	0.073922755	0.009320997
NF1-P53	2	EGFR Y1068	0.436628558	4.061619405	0.107501101	0.009320997
NF1-P53	2	PKCz-L T410/T403	0.429872432	3.905731493	0.110061952	0.011659665
NF1-P53	2	Cofilin S3	0.419253559	3.722958236	0.112613017	0.015416075
NF1-P53	2	ERBB2 Y1248	0.403440708	3.682397883	0.109559239	0.015444474
NF1-P53	2	AMPKa T172	0.294335447	3.665033139	0.080309082	0.015444474
NF1-P53	2	SGK1 S78	0.78686947	3.243479394	0.242600422	0.032293889
NF1-P53	2	PKCa-BII T638/T641.1	0.411619851	3.196770414	0.128761155	0.032500124
NF1-P53	2	FOXO3a S256	0.180072557	3.191579087	0.056421148	0.032500124
NF1-P53	2	SRC Y527	0.393073974	3.129259805	0.125612445	0.03361802
NF1-P53	2	PYK2 Y402	0.611115205	3.129124953	0.195299074	0.03361802
NF1-P53	2	NFKB p65 S536	0.285184916	3.091711009	0.092241777	0.03467691
NF1-P53	2	ELK-1 S383	0.241889867	3.033426738	0.079741457	0.037253839
NF1-P53	2	STAT3 Y705	0.264961472	2.993820517	0.088502791	0.038371634
NF1-P53	2	ATR S428	0.333482114	2.979610307	0.111921386	0.038371634
NF1-P53	2	ERBB3 Y1197	0.209400955	2.921352907	0.071679445	0.040882897
NF1-P53	2	4EBP1 T37/46	0.232743611	2.910281191	0.079972895	0.040882897
NF1-P53	2	cKit Y719	0.17288198	2.882589378	0.059974543	0.041716331
NF1-P53	2	HSP27 S82	0.546905289	2.625662433	0.208292308	0.065387908
NF1-P53	2	EGFR Y1148	0.300051483	2.338387089	0.128315575	0.107877848
NF1-P53	2	PAK2 S20	0.421223599	2.311456784	0.182232954	0.110081135
NF1-P53	2	JAK1 Y1022/Y1023	0.226719736	2.282915398	0.099311493	0.112739035
NF1-P53	2	elF4G S1108	0.447932511	2.190751451	0.204465235	0.120340855
NF1-P53	2	LIMK1 T508/LIMK2 T505	−0.445656431	−2.166426331	0.205710402	0.120340855
NF1-P53	2	ATF2 T69/71	−4.483851066	−2.156060658	2.079649777	0.120340855
NF1-P53	2	Ephrin A3 Y799/A4 Y799/A5 Y833	0.219927822	2.155791819	0.102017189	0.120340855
NF1-P53	2	AXL Y702	0.16013891	2.144282221	0.074681825	0.120340855
NF1-P53	2	Chk1 S345	−0.867735767	−2.141068475	0.405281651	0.120340855
NF1-P53	2	EGFR Y1173	0.594358949	2.125542156	0.279626987	0.120340855
NF1-P53	2	p90RSK S380	0.206042768	2.123177847	0.097044516	0.120340855
NF1-P53	2	eNOS S1177	0.349582937	2.117582399	0.165085872	0.120340855
NF1-P53	2	S6RP S235/S236	−0.438272388	−2.111031895	0.207610501	0.120340855
NF1-P53	2	SAPK/JNK T183/Y185	0.226261372	2.047154124	0.110524835	0.130212957
NF1-P53	2	JAK2 Y1007	0.339950076	2.035897464	0.166977995	0.130212957
NF1-P53	2	SUMO2/3 total	0.188737227	2.031289417	0.092914985	0.130212957
NF1-P53	2	Raf S259	0.350285464	1.942261354	0.180349294	0.149278961
NF1-P53	2	PAK1 T423/PAK2 T402	0.112535305	1.900196996	0.059222968	0.15750216
NF1-P53	2	4EBP1 T70	0.225779621	1.866683435	0.120952282	0.162348866
NF1-P53	2	SEK1/MKK4 S80	0.270497053	1.86055151	0.145385415	0.162348866
NF1-P53	2	PKCa-BII T638/T641	0.311119633	1.766824766	0.176089695	0.187145595
NF1-P53	2	ALK Y1586	0.132000358	1.730004546	0.076300585	0.195442004
NF1-P53	2	MET Y1234/Y1235	0.136078743	1.712320537	0.079470368	0.195442004
NF1-P53	2	Acetyl CoAC S79	0.192622266	1.709275461	0.112692349	0.195442004
NF1-P53	2	cKit Y719.1	−0.095457855	−1.55748383	0.061289789	0.247059458
NF1-P53	2	Insulin Rec beta total	0.243733375	1.522261582	0.160112676	0.257181237
NF1-P53	2	PTEN total	0.271065562	1.481409013	0.182978205	0.270097317
NF1-P53	2	ATF2 T71	−0.170476165	−1.469068285	0.116043731	0.270969174
NF1-P53	2	mTOR S2448	0.236164957	1.413586657	0.167067902	0.291137456
NF1-P53	2	cAbl T735	0.233992294	1.383691566	0.169107263	0.300280774
NF1-P53	2	AMPKb1 S108	0.189719744	1.351725949	0.140353704	0.306315814
NF1-P53	2	IRS1 S612	0.147356104	1.344166693	0.109626362	0.306315814
NF1-P53	2	PRK1 T774/PRK2 T816	0.492186186	1.335865163	0.368440018	0.306315814
NF1-P53	2	AMPKa1 S485	0.173551816	1.331212781	0.130371206	0.306315814
NF1-P53	2	PRAS40 T246	0.210379849	1.315503715	0.159923417	0.308949708
NF1-P53	2	ERBB3 Y1289	0.239761306	1.306480341	0.183516964	0.308949708
NF1-P53	2	4EBP1 S65	0.120729161	1.284716269	0.093973404	0.314814533
NF1-P53	2	STAT1 Y701	−0.146905554	−1.261254191	0.116475771	0.321611473
NF1-P53	2	SRC Family Y416	−0.172644004	−1.100542592	0.156871715	0.401776139
NF1-P53	2	FOXO1 S256	0.161319084	1.087436909	0.148347994	0.403766118
NF1-P53	2	B-RAF S445	0.086568042	1.036768481	0.083497949	0.427878345
NF1-P53	2	IGF1R Y1135/Y1136-IR Y1150/Y1151	0.086052305	1.016182652	0.084681927	0.434472241
NF1-P53	2	STAT4 Y693	0.192308579	0.967008494	0.198869586	0.458785274
NF1-P53	2	MCSFR Y732	0.136118264	0.95601657	0.142380654	0.459650554
NF1-P53	2	EGFR	0.096651035	0.938840801	0.102947203	0.463832839
NF1-P53	2	p38 MAPK T180/Y182	−0.066818407	−0.922426986	0.072437611	0.463832839
NF1-P53	2	cABL Y245	0.113245687	0.920949879	0.122966178	0.463832839
NF1-P53	2	p70S6K T389	−0.139200662	−0.898884989	0.154859257	0.47177771
NF1-P53	2	ERK 1/2 total	0.129601234	0.777377268	0.166716007	0.538749885
NF1-P53	2	FRS2a Y436	0.101692386	0.777330153	0.130822644	0.538749885
NF1-P53	2	ERBB3 total	0.157347755	0.733829217	0.214420128	0.561931424
NF1-P53	2	Aurora AT288/BT232/CT198	0.052414213	0.723617075	0.072433633	0.561931424
NF1-P53	2	ERBB4 total	−0.085129277	−0.714727449	0.119107329	0.561931424
NF1-P53	2	Paxillin Y118	0.40248955	0.667079652	0.603360557	0.588378613
NF1-P53	2	HSP90a T5/T7	0.127216857	0.62179464	0.204596259	0.613628774
NF1-P53	2	FAK Y576/Y577	0.076011002	0.453037429	0.167780844	0.732402293
NF1-P53	2	PAK1 S199/S204-PAK2 S192/S197	0.075967466	0.413771172	0.183597774	0.754794122
NF1-P53	2	ATPcitrate Lyase S454	0.089061158	0.391078908	0.227731938	0.764192257
NF1-P53	2	S6RP S240/S244	−0.038460057	−0.368639052	0.10432985	0.77335535
NF1-P53	2	PTEN S380	−0.021371962	−0.27591531	0.077458411	0.837988619
NF1-P53	2	IGF1R Y1131/IR Y1146	0.043792192	0.236068329	0.185506424	0.860742894
NF1-P53	2	FOXO1 T24/FOXO3a T32	0.0214416	0.182521197	0.117474574	0.894284957
NF1-P53	2	PDK1 S241	−0.009597387	−0.103406735	0.092812011	0.948038149
NF1-P53	2	MST1 T183/MST2 T180	−0.008415726	−0.0901971	0.093303735	0.948751765
NF1-P53	2	p70S6K S371	−0.013011528	−0.06305636	0.20634759	0.960437771
NF1-P53	2	STAT5 Y694	0.001268889	0.015436168	0.082202321	0.987932164
NF1-MET	48	AMPKa1 S485	0.37796374	4.946710116	0.076407093	0.018034171
NF1-MET	48	PAK1 S199/S204-PAK2 S192/S197	−0.40007899	−4.819039561	0.083020483	0.018034171
NF1-MET	48	C-RAF S338	0.33997879	4.054645186	0.083849209	0.0477197
NF1-MET	48	MET Y1234/Y1235	−0.138757341	−3.791211758	0.036599734	0.058400691
NF1-MET	48	cKit Y719	−0.205340704	−3.66161783	0.056079229	0.059572304
NF1-MET	48	PTEN S380	−0.139448487	−3.091750027	0.045103416	0.110263618
NF1-MET	48	PYK2 Y402	−0.221909975	−3.05359782	0.072671645	0.110263618
NF1-MET	48	IGF1R beta total	−1.190846864	−3.038981056	0.391857285	0.110263618
NF1-MET	48	Paxillin Y118	−1.072641782	−3.026197152	0.354452049	0.110263618
NF1-MET	48	ATF2 T69/71	0.853900834	2.538571829	0.336370562	0.239282081
NF1-MET	48	EGFR Y1068	0.568572377	2.509580101	0.226560761	0.239282081
NF1-MET	48	HSP27 S82	−0.414401695	−2.319399559	0.178667661	0.312171404
NF1-MET	48	B-RAF S445	−0.141869056	−2.147974306	0.066047836	0.3283577
NF1-MET	48	STAT4 Y693	−0.217067811	−2.146893135	0.101107879	0.3283577
NF1-MET	48	JAK1 Y1022/Y1023	−0.140156149	−2.123675315	0.065996976	0.3283577
NF1-MET	48	AXL Y702	−0.19260434	−2.060228379	0.093486888	0.3283577
NF1-MET	48	ATF2 T71	0.308000079	2.041506752	0.150868999	0.3283577
NF1-MET	48	FOXO1 T24/FOXO3a T32	0.180279826	2.009693407	0.089705139	0.3283577
NF1-MET	48	SGK1 S78	0.200769041	2.007064195	0.1000312	0.3283577
NF1-MET	48	PRAS40 T246	0.126670824	1.988108033	0.063714256	0.3283577
NF1-MET	48	ATR S428	0.117317044	1.982296893	0.059182378	0.3283577
NF1-MET	48	PKCz-L T410/T403	0.383182032	1.88883495	0.202866869	0.359392363
NF1-MET	48	FRS2a Y436	−0.136031345	−1.870908783	0.0727087	0.359392363
NF1-MET	48	EGFR	−0.122447681	−1.85483888	0.066015265	0.359392363
NF1-MET	48	ERBB3 Y1289	0.079814489	1.826726654	0.043692629	0.362257368
NF1-MET	48	AKT S473	−0.179936555	−1.716076269	0.104853472	0.416719138
NF1-MET	48	LIMK1 T508/LIMK2 T505	0.223147981	1.688686794	0.132142907	0.416719138
NF1-MET	48	4EBP1 S65	−0.102675468	−1.678317444	0.061177621	0.416719138
NF1-MET	48	cAbl T735	0.08577078	1.650350497	0.051971251	0.421685598
NF1-MET	48	ERK1/2 T202/Y204	−0.185725311	−1.500794006	0.123751367	0.52142927
NF1-MET	48	eNOS S1177	−0.0786173	−1.413602386	0.055614861	0.566481485
NF1-MET	48	YAP S127	−0.119722462	−1.408627137	0.084992301	0.566481485
NF1-MET	48	MCSFR Y732	0.11587647	1.352506833	0.085675331	0.583643249
NF1-MET	48	Aurora AT288/BT232/CT198	0.079586916	1.35007624	0.058949942	0.583643249
NF1-MET	48	ERBB3 Y1197	0.077650264	1.332247073	0.058285183	0.583643249
NF1-MET	48	FAK Y576/Y577	−0.131890129	−1.310767883	0.100620507	0.586546235
NF1-MET	48	ELK-1 S383	−0.070712657	−1.20347983	0.058756827	0.644081422
NF1-MET	48	CRKII Y221	0.100750104	1.20012136	0.08394993	0.644081422
NF1-MET	48	cABL Y245	0.074552422	1.191318343	0.062579765	0.644081422
NF1-MET	48	GSK3aB S21/S9	0.096144411	1.178779481	0.081562678	0.644081422
NF1-MET	48	ERK 1/2 total	−0.045697609	−1.129934619	0.040442701	0.674948012
NF1-MET	48	Ephrin A3 Y799/A4 Y799/A5 Y833	0.060344409	1.079496162	0.055900531	0.695618534
NF1-MET	48	RAS-GRF1 S916	0.063022824	1.050403438	0.059998684	0.695618534
NF1-MET	48	AKT T308	−0.070924821	−1.048968893	0.067613846	0.695618534
NF1-MET	48	Chk1 S345	0.16047593	1.024497505	0.156638673	0.695618534
NF1-MET	48	PDK1 S241	0.109884181	1.020968175	0.10762743	0.695618534
NF1-MET	48	p90RSK S380	0.073311803	1.003218825	0.073076582	0.695618534
NF1-MET	48	mTOR S2448	−0.102220137	−0.997490811	0.102477272	0.695618534
NF1-MET	48	PRK1 T774/PRK2 T816	0.22716092	0.980105562	0.231771891	0.697952328
NF1-MET	48	p38 MAPK T180/Y182	0.073336447	0.908135058	0.080755	0.75402912
NF1-MET	48	p70S6K S371	0.079345336	0.889890787	0.089163005	0.757397037
NF1-MET	48	Acetyl CoAC S79	0.043279678	0.833547946	0.051922242	0.789218168
NF1-MET	48	ERBB3 total	0.078842399	0.82899535	0.095105961	0.789218168
NF1-MET	48	cKit Y719.1	−0.04353576	−0.810352496	0.053724472	0.793382834
NF1-MET	48	Insulin Rec beta total	0.076941321	0.75785152	0.101525588	0.822495297
NF1-MET	48	JAK2 Y1007	0.061968239	0.743313017	0.083367622	0.822495297
NF1-MET	48	PAK2 S20	−0.041619237	−0.732194507	0.056841777	0.822495297
NF1-MET	48	elF4E S209	0.061350183	0.724686088	0.084657597	0.822495297
NF1-MET	48	IGF1R Y1131/IR Y1146	−0.065404199	−0.697273547	0.093799914	0.835006176
NF1-MET	48	ERBB4 total	−0.055801468	−0.660584693	0.084472845	0.835006176
NF1-MET	48	STAT1 Y701	−0.090622465	−0.659315913	0.137449231	0.835006176
NF1-MET	48	PDGFRb Y751	0.040637743	0.657080039	0.061845956	0.835006176
NF1-MET	48	PAK1 T423/PAK2 T402	0.030554298	0.607107384	0.050327666	0.860421265
NF1-MET	48	IRS1 S612	0.048608617	0.593295138	0.08192991	0.860421265
NF1-MET	48	Cofilin S3	0.033663051	0.591051049	0.056954558	0.860421265
NF1-MET	48	p70S6K T389	−0.053843581	−0.57520891	0.093607001	0.862894592
NF1-MET	48	STAT5 Y694	−0.040637038	−0.486271077	0.083568693	0.877044712
NF1-MET	48	FOXO1 S256	0.020685928	0.480263471	0.04307204	0.877044712
NF1-MET	48	p90RSK T359/S363	0.085342308	0.458611917	0.186088291	0.877044712
NF1-MET	48	elF4G S1108	0.028573198	0.451725765	0.063253417	0.877044712
NF1-MET	48	ERBB2 Y1248	0.022343103	0.450190976	0.049630277	0.877044712
NF1-MET	48	4EBP1 T37/46	−0.031266332	−0.428262034	0.073007481	0.877044712
NF1-MET	48	FGFR Y653/Y654	0.024775964	0.413252934	0.05995351	0.877044712
NF1-MET	48	S6RP S240/S244	0.087083602	0.412707785	0.211005474	0.877044712
NF1-MET	48	PTEN total	0.033659637	0.408980929	0.082301239	0.877044712
NF1-MET	48	MEK1/2 S217/S221	0.025026013	0.406845664	0.0615123	0.877044712
NF1-MET	48	SEK1/MKK4 S80	0.031709205	0.376866596	0.08413907	0.877044712
NF1-MET	48	EGFR Y1148	−0.042305995	−0.368430913	0.114827484	0.877044712
NF1-MET	48	SAPK/JNK T183/Y185	−0.025225966	−0.359730068	0.070124708	0.877044712
NF1-MET	48	SUMO2/3 total	0.021384676	0.358908705	0.059582494	0.877044712
NF1-MET	48	PKCa-BII T638/T641.1	0.032736948	0.358506867	0.091314704	0.877044712
NF1-MET	48	EGFR Y1173	−0.018738898	−0.34580907	0.054188568	0.877044712
NF1-MET	48	Raf S259	−0.032928323	−0.345649482	0.095265073	0.877044712
NF1-MET	48	ATPcitrate Lyase S454	0.040289704	0.322310662	0.125002702	0.886868641
NF1-MET	48	FOXO3a S256	−0.011309756	−0.281400429	0.04019097	0.9119234
NF1-MET	48	IGF1R Y1135/Y1136-IR Y1150/Y1151	−0.018267631	−0.258864553	0.0705683	0.920831358
NF1-MET	48	S6RP S235/S236	0.045152057	0.236236347	0.191130863	0.922691109
NF1-MET	48	AMPKa T172	−0.014678808	−0.219498568	0.066874278	0.922691109
NF1-MET	48	MST1 T183/MST2 T180	0.011988399	0.211474763	0.056689504	0.922691109
NF1-MET	48	SRC Y527	0.019042718	0.207657342	0.091702598	0.922691109
NF1-MET	48	STAT3 Y705	0.010631514	0.158180199	0.067211407	0.939348158
NF1-MET	48	TYK2 Y1054/Y1055	0.011323118	0.156856502	0.072187748	0.939348158
NF1-MET	48	HSP90a T5/T7	0.006759779	0.150928521	0.044787949	0.939348158
NF1-MET	48	NFKB p65 S536	0.009859336	0.101787287	0.096862159	0.969489151
NF1-MET	48	AMPKb1 S108	0.005774302	0.083278346	0.06933738	0.974302478
NF1-MET	48	PKCa-BII T638/T641	−0.003882564	−0.059201386	0.065582313	0.974469857
NF1-MET	48	4EBP1 T70	−0.002416504	−0.057837829	0.041780675	0.974469857
NF1-MET	48	SRC Family Y416	−0.006148277	−0.039082924	0.15731363	0.979328004
NF1-MET	48	ALK Y1586	−1.96E-06	−4.63E-05	0.04231125	0.999963801
NF1-P53	48	p70S6K S371	−1.188965716	−14.70413955	0.080859251	5.37E-07
NF1-P53	48	Cofilin S3	0.935505427	8.510809545	0.109919676	0.000104186
NF1-P53	48	STAT3 Y705	0.642420785	8.15328966	0.078792832	0.000107972
NF1-P53	48	AKT T308	−0.815153411	−7.176297718	0.113589687	0.00029118
NF1-P53	48	PKCz-L T410/T403	−1.023016875	−6.821386732	0.14997198	0.000381664
NF1-P53	48	CRKII Y221	0.730429189	6.413123032	0.11389602	0.000572755
NF1-P53	48	PRK1 T774/PRK2 T816	−0.916501363	−5.934462616	0.154437128	0.001006921
NF1-P53	48	cABL Y245	0.35898297	5.507073199	0.0651858	0.001718896
NF1-P53	48	AXL Y702	0.323622067	5.245046716	0.061700512	0.002331113
NF1-P53	48	PDGFRb Y751	0.324597283	4.420434338	0.073431083	0.008434293
NF1-P53	48	HSP90a T5/T7	−0.356350306	−4.275722489	0.083342712	0.009401693
NF1-P53	48	SAPK/JNK T183/Y185	0.399355681	4.254391705	0.093869044	0.009401693
NF1-P53	48	ATPcitrate Lyase S454	0.954435669	4.009396519	0.238049707	0.013409643
NF1-P53	48	ERBB3 Y1289	−0.294289464	−3.814946532	0.077141177	0.017668328
NF1-P53	48	SGK1 S78	−0.551031351	−3.645740019	0.151143896	0.021128552
NF1-P53	48	PAK2 S20	−0.303591378	−3.629206001	0.083652286	0.021128552
NF1-P53	48	PKCa-BII T638/T641	−0.731892773	−3.609761183	0.202753793	0.021128552
NF1-P53	48	ATR S428	0.254886444	3.519007289	0.072431349	0.022329025
NF1-P53	48	EGFR Y1068	0.328859293	3.509537826	0.093704445	0.022329025
NF1-P53	48	Aurora AT288/BT232/CT198	−0.217101752	−3.438953087	0.063130187	0.022329025
NF1-P53	48	p38 MAPK T180/Y182	0.437365607	3.428799436	0.127556486	0.022329025
NF1-P53	48	ERBB3 total	−0.382293598	−3.420964573	0.111750236	0.022329025
NF1-P53	48	AKT S473	−0.271305264	−3.414708506	0.079451954	0.022329025
NF1-P53	48	ATF2 T71	0.523404131	3.362957619	0.15563804	0.023537137
NF1-P53	48	4EBP1 T37/46	0.259673818	3.087643825	0.084100963	0.037339208
NF1-P53	48	PKCa-BII T638/T641.1	0.324103867	3.069849701	0.105576461	0.037339208
NF1-P53	48	MST1 T183/MST2 T180	−0.245253836	−2.971174467	0.082544407	0.043163301
NF1-P53	48	ELK-1 S383	0.194285926	2.686687406	0.072314303	0.070421213
NF1-P53	48	ATF2 T69/71	4.92824451	2.666931937	1.847907868	0.070513448
NF1-P53	48	IGF1R Y1131/IR Y1146	−0.206288428	−2.529022478	0.081568444	0.087817939
NF1-P53	48	S6RP S235/S236	−1.284256801	−2.467278564	0.520515526	0.091819324
NF1-P53	48	PYK2 Y402	−0.236371173	−2.463773255	0.095938688	0.091819324
NF1-P53	48	FRS2a Y436	0.195977734	2.452512663	0.079908959	0.091819324
NF1-P53	48	ERK1/2 T202/Y204	−0.275334191	−2.376040759	0.115879406	0.102426463
NF1-P53	48	GSK3aB S21/S9	0.195463433	2.285629702	0.085518417	0.117184977
NF1-P53	48	AMPKa1 S485	0.502817468	2.159942433	0.232792069	0.140846941
NF1-P53	48	FAK Y576/Y577	−0.281971411	−2.152030979	0.131025721	0.140846941
NF1-P53	48	MCSFR Y732	−0.289477295	−2.048184043	0.141333634	0.164836444
NF1-P53	48	elF4E S209	−0.259668428	−1.94110306	0.133773643	0.191172735
NF1-P53	48	S6RP S240/S244	−0.634754883	−1.934064596	0.328197354	0.191172735
NF1-P53	48	PRAS40 T246	−0.208445063	−1.862091972	0.111941336	0.211181199
NF1-P53	48	ALK Y1586	−0.086544806	−1.800294748	0.048072576	0.226392758
NF1-P53	48	AMPKa T172	0.122843009	1.793466054	0.06849475	0.226392758
NF1-P53	48	Raf S259	−0.158437654	−1.75036374	0.090516988	0.237983813
NF1-P53	48	cKit Y719.1	−0.127652185	−1.684118643	0.07579762	0.260009307
NF1-P53	48	PTEN total	−0.63558934	−1.57251607	0.404186228	0.305673729
NF1-P53	48	SRC Y527	0.152181892	1.515055344	0.100446425	0.315011785
NF1-P53	48	C-RAF S338	0.152179573	1.499208411	0.101506616	0.315011785
NF1-P53	48	PAK1 S199/S204-PAK2 S192/S197	−0.369317218	−1.494488474	0.247119482	0.315011785
NF1-P53	48	TYK2 Y1054/Y1055	0.078381165	1.488168541	0.052669548	0.315011785
NF1-P53	48	JAK1 Y1022/Y1023	−0.125996483	−1.48726241	0.084717049	0.315011785
NF1-P53	48	ERBB3 Y1197	−0.117075464	−1.477708348	0.079227721	0.315011785
NF1-P53	48	SRC Family Y416	0.127634129	1.45584991	0.08766984	0.319492542
NF1-P53	48	FGFR Y653/Y654	0.151703263	1.436909197	0.105576095	0.319492542
NF1-P53	48	PDK1 S241	0.088973018	1.433392793	0.062071624	0.319492542
NF1-P53	48	SUMO2/3 total	0.103132847	1.407136185	0.073292726	0.326997325
NF1-P53	48	Ephrin A3 Y799/A4 Y799/A5 Y833	−0.180092097	−1.277949833	0.140922666	0.386887774
NF1-P53	48	B-RAF S445	0.100969172	1.268238144	0.079613732	0.386887774
NF1-P53	48	cAbl T735	−0.125213221	−1.263601749	0.099092314	0.386887774
NF1-P53	48	p70S6K T389	−0.153886111	−1.24316367	0.123785882	0.392281236
NF1-P53	48	Chk1 S345	0.630391381	1.228628023	0.513085628	0.394308818
NF1-P53	48	p90RSK T359/S363	0.13560333	1.067531949	0.127025079	0.490070134
NF1-P53	48	STAT4 Y693	−0.115635787	−1.040006984	0.11118751	0.500880595
NF1-P53	48	Insulin Rec beta total	0.078408158	1.029315133	0.076175075	0.500880595
NF1-P53	48	ERBB2 Y1248	−0.09450299	−0.981403598	0.096293707	0.52687515
NF1-P53	48	FOXO3a S256	−0.045673628	−0.940066001	0.048585555	0.548815244
NF1-P53	48	MEK1/2 S217/S221	0.15845465	0.909935188	0.174138391	0.562851827
NF1-P53	48	MET Y1234/Y1235	−0.098355252	−0.882576555	0.111441044	0.568244527
NF1-P53	48	STAT1 Y701	−0.3231581	−0.88045342	0.367035998	0.568244527
NF1-P53	48	PAK1 T423/PAK2 T402	−0.050512557	−0.828956087	0.060935142	0.598874899
NF1-P53	48	cKit Y719	0.052637198	0.81258753	0.064777266	0.60294147
NF1-P53	48	mTOR S2448	−0.079164867	−0.770668553	0.102722327	0.618645183
NF1-P53	48	EGFR Y1148	0.103185775	0.758926123	0.135962872	0.618645183
NF1-P53	48	Paxillin Y118	−0.196136641	−0.743697607	0.263731708	0.618645183
NF1-P53	48	PTEN S380	−0.069940382	−0.741307325	0.094347351	0.618645183
NF1-P53	48	JAK2 Y1007	0.053117456	0.737827661	0.071991685	0.618645183
NF1-P53	48	4EBP1 S65	0.077175391	0.680140851	0.113469718	0.654984852
NF1-P53	48	IRS1 S612	0.063683041	0.658489792	0.096710749	0.66350465
NF1-P53	48	ERK 1/2 total	−0.044954448	−0.644743217	0.069724578	0.665841259
NF1-P53	48	AMPKb1 S108	−0.107233733	−0.626869931	0.171062174	0.671449617
NF1-P53	48	Acetyl CoAC S79	0.054099695	0.595576941	0.090835779	0.687640734
NF1-P53	48	ERBB4 total	−0.060806728	−0.520620945	0.116796546	0.739097423
NF1-P53	48	YAP S127	0.029101588	0.47467031	0.061309055	0.76768547
NF1-P53	48	EGFR	−0.056524183	−0.443436335	0.127468542	0.784228074
NF1-P53	48	SEK1/MKK4 S80	−0.068063891	−0.427633572	0.15916405	0.787987731
NF1-P53	48	4EBP1 T70	0.037997429	0.405895785	0.093613756	0.796631623
NF1-P53	48	elF4G S1108	−0.033177858	−0.384966873	0.086183669	0.804289504
NF1-P53	48	LIMK1 T508/LIMK2 T505	−0.035473615	−0.374082289	0.094828374	0.804289504
NF1-P53	48	EGFR Y1173	−0.122134664	−0.351948796	0.347023958	0.813169445
NF1-P53	48	eNOS S1177	−0.019962912	−0.33435334	0.059706033	0.818325836
NF1-P53	48	FOXO1 T24/FOXO3a T32	−0.06477384	−0.320081991	0.202366399	0.820782828
NF1-P53	48	HSP27 S82	0.669621519	0.3086704	2.169373926	0.820957233
NF1-P53	48	RAS-GRF1 S916	−0.021866434	−0.248226928	0.088090498	0.860332418
NF1-P53	48	IGF1R Y1135/Y1136-IR Y1150/Y1151	−0.009133196	−0.180227818	0.050675841	0.905751821
NF1-P53	48	STAT5 Y694	0.013476121	0.167466405	0.080470592	0.906439401
NF1-P53	48	p90RSK S380	0.006947483	0.118612177	0.058573097	0.935922833
NF1-P53	48	NFKB p65 S536	−0.009933543	−0.081245524	0.12226573	0.95186899
NF1-P53	48	IGF1R beta total	−0.01082882	−0.064614954	0.167589995	0.95186899
NF1-P53	48	FOXO1 S256	0.013225355	0.061644904	0.214540926	0.95186899

### p53 loss drives mTOR Dependency in MPNSTs

**Fig. 5:**
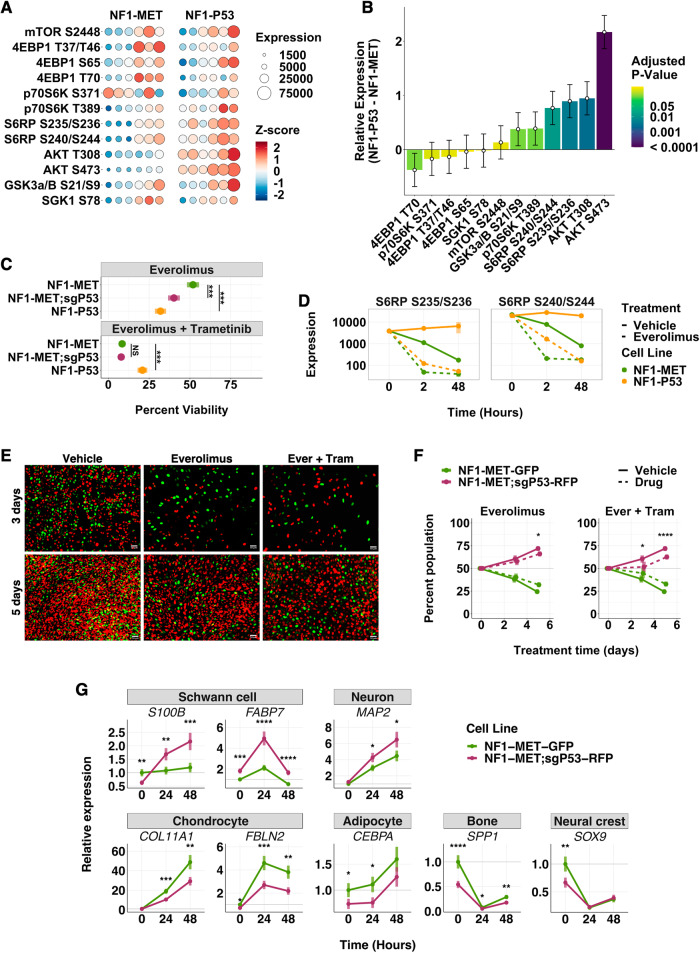
p53 deficiency induces mTOR dependency. **A** mTORC1/mTORC2 phospho-protein expression (size) and z - score (color) of NF1-MET and NF1-P53 tumors (6 animals/group). **B** Contrast estimates +/− SE of mTORC1/mTORC2 phospho-protein expression in NF1-P53 tumors compared to NF1-MET tumors. Color indicates *P*-value. **C** Percent viability of NF1-MET, NF1-MET;sgP53, and NF1-P53 cells after 72 h of everolimus (20 nM) or combination (everolimus 20 nM, trametinib 40 nM) treatment. **D** Phospho-S6RP expression measured over time by RPPA after vehicle (DMSO) or everolimus (100 nM) treatment. Images (**E**) and flow cytometry analysis (**F**) of GFP labeled NF1-MET and RFP labeled NF1-MET;sgP53 cells after 3 and 5 days of treatment with vehicle (DMSO), everolimus (20 nM), or combination everolimus (20 nM) and trametinib (40 nM). **G** Expression (relative to the housekeeping gene PPIA) of cell fate markers by qRT-PCR upon treatment with combination everolimus (20 nM) and trametinib (40 nM). See also Supplementary Fig. 9E. *p < 0.05, **p < 0.01, ***p < 0.001, ****p < 0.0001.

AKT activation was consistently elevated and sustained in p53-deficient MPNST cell lines and is targetable therapeutically, either directly or via its downstream effectors. To determine the scope of AKT/mTOR pathway activation in vivo, we assessed the phosphorylation status of mTORC1 and mTORC2 pathway effectors in MPNST tumorgrafts by RPPA. Globally, phosphorylation was significantly increased in NF1-P53 tumors compared to NF1-MET tumors (*p*-value = 0.0034) (Fig. 5A), with increased activation of 7/12 phosphosites in NF1-P53 tumors (Fig. 5B). AKT and S6RP phosphorylation were significantly increased in the NF1-P53 tumors (Fig. 5B), suggesting increased dependency on the AKT/mTOR pathway. Regardless of p53 status, treatment with the AKT inhibitor, afuresertib, had no effect on the MPNST cell lines, either as a single agent or in combination with trametinib (Supplementary Fig. 7A, B). However, treatment with the mTOR inhibitor, everolimus, significantly decreased viability in the p53-deficient cell lines compared to the p53-intact cells (Fig. 5C). This enhanced inhibition of the p53-deficient cells was observed even though everolimus strongly inhibited downstream pS6RP regardless of p53 status (Fig. 5D). Further, combination therapy of mTOR (everolimus) and MEK (trametinib) inhibition reversed clonal selection for p53 loss (Fig. 5E, F).

As combination mTOR and MEK inhibition was so effective in inhibiting MPNST cell growth, we next asked whether treatment reversed the nuclear localization of MET leading to global downregulation of MAPK and AKT/mTOR signaling. Unexpectedly, combination everolimus and trametinib actually induced ligand-independent MET localization specifically in the NF1-MET;sgP53 cells compared to the parental line (Supplementary Fig. 8A). Moreover, treatment with everolimus or the dual PI3K/mTOR inhibitor BEZ235 induced stronger compensatory ERK and AKT activation in NF1-P53 cells, consistent with increased MET activation (Supplementary Fig. 8B). These data reinforce the role of p53 in regulating MET localization and effector signaling in response to diverse stimuli.

We next tested whether the excessive AKT activation induced by everolimus resulted in p53-independent oncogene induced apoptosis or senescence in p53 null MPNST cells [59, 60]. Combination everolimus and trametinib treatment did induce apoptosis in the NF1-MET;sgP53 cells compared to the parental cell line, however, the difference was minor (Supplementary Fig. 9A, B). We also did not observe senescence based on expression of senescence-associated secretory phenotype markers (Supplementary Fig. 9C), or by staining with the senescence marker beta-galactosidase after 7 days of drug treatment (Supplementary Fig. 9D).

We evaluated whether differentiation of MPNST cells was impacted by combined everolimus and trametinib treatment. We observed that mTOR and MEK inhibition significantly increased expression of the Schwann cell markers *S100B* and *FABP7* in NF1-MET;sgP53 but not NF1-MET cells (Fig. 5G). We also examined differentiation marker expression of neuron, chondrocyte, adipocyte, bone, kidney, endothelial, and muscle cells, as well as the multipotency marker *SOX9*, which is expressed in neural crest cells. Combined MEK and mTOR inhibition resulted in increase of the neuronal marker *MAP2* in NF1-MET;sgP53 cells, while chondrocyte and adipocyte differentiation were strongly induced in NF1-MET cells (Fig. 5G). Interestingly, both kidney and muscle markers were strongly induced by treatment in both NF1-MET and NF1-MET;sgP53 line (Supplementary Fig. 9E). Consistent with the induction of multiple differentiation pathways upon mTOR and MEK inhibition, the multipotency marker *SOX9* was decreased in both cell lines (Fig. 5G). These results suggest that lineage differentiation is altered by mTOR and MEK inhibition, with p53-deficient MPNST cells most sensitive to shifts back towards differentiated Schwann cell states.

## Discussion

Resistance to both chemotherapy and targeted kinase inhibition in NF1-related MPNSTs is a daunting clinical challenge. Although MPNSTs harbor complex genomic alterations, copy number gains of RTKs, such as MET, PDGFRα, and EGFR are commonly detected [34, 61]. Moreover, autocrine MET-HGF signaling has been demonstrated to promote acquired resistance to MEK inhibition [61]. Previously, we observed that p53 status impacted the therapeutic response to combined MET and MEK inhibition in *Met*-amplified MPNST tumorgrafts. Even though it is known that p53 is an independent predictor of poor survival and poor response to neoadjuvant chemotherapy in MPNSTs [28, 31], how p53 function influences MPNST therapeutic response is not fully understood. We examined whether p53 has a ‘non-canonical’ function and discovered a novel role for p53 in modulating kinome adaptations to targeted therapy. A comprehensive transcriptional and phosphoproteomic analysis revealed multiple mechanisms of resistance, including deregulation of MET stability, localization, and effector activation.

We used 3 genetic models of p53-deficient, *Met*-amplified MPNST cells to examine targeted therapy resistance. The NF1-p53 cell line was generated from a murine MPNST driven by loss of *Trp53* and *Nf1*, with *Met* amplification occurring spontaneously, while the NF1-MET cell line was driven by amplification of *Met* in the context of *Nf1* loss [34], with spontaneous deletion of *Cdkn2a*. We further used Crispr-Cas9 to generate a p53-deficient isogenic cell line, NF1-MET;sgP53, from the NF1-MET cells to identify p53-specific regulation of drug response. In general, the p53-deficient cells demonstrated similar phenotypes, although in several experiments drug responses were exaggerated in the NF1-P53 cells compared to the NF1-MET;sgP53 cells. These stronger phenotypes may be caused by additional genomic alterations caused by early loss of p53, compared to our isogenic model in which p53 loss was a late event. Interestingly, *Cdkn2a* was wildtype in NF1-P53 cells, but deleted in NF1-MET cells. In patients with NF1, *CDKN2A* is an early loss to promote atypical neurofibroma [41], while additional loss of tumor suppressors (e.g. *TP53*) and/or amplification of RTKs (e.g. *MET* or *EGFR*) is required for malignant transformation to MPNST [8]. Understanding how the stepwise timing and clonal evolution of MPNST dictates drug response is critical for developing new therapies.

p53-deficient MPNSTs exhibited increased baseline MET activation suggesting that p53 loss disinhibits amplified MET signaling, an effect that is exaggerated in response to HGF. To determine if p53 is a key driver of clonal selection and drug resistance in MPNSTs, we performed clonal competition assays and discovered that p53-deficient cells had a significant growth advantage in response to MET or MEK inhibition. RNA-seq analysis revealed that p53 regulates expression of several genes involved in MET localization and receptor turnover, resulting in altered signaling kinetics and effector activation. p53 loss resulted in decreased *Cblb* expression, an E3 ubiquitin ligase that targets both MET [52, 53] and EGFR [56] for lysosomal degradation. p53 loss also induced *Prkce* expression, which is required for nuclear MET translocation [55], and increased activation of AKT, which is required for nuclear EGFR translocation [62]. Collectively, these data suggest a model in which p53 loss causes increased MET localization to the nucleus. In breast and ovarian cancers nuclear MET results in altered effector activation leading to increased calcium signaling [63], PARP activation [51], and YAP-dependent transcriptional activation [50] to promote drug resistance and invasion/metastasis. Interestingly, nuclear MET also induces sustained STAT3 activation [58], which occurred in response to HGF in our non-isogenic NF1-P53 cell line. In oncogene-addicted cancers, a positive feedback look that promotes STAT3 activation induces resistance to targeted therapy [64]. As the majority of MPNSTs demonstrate loss of function of p53, our findings may partially explain the unexpected failure of the EGFR inhibitor erlotinib in a previous clinical trial in MPNST [57]. Overall, these data indicate that p53 status should be evaluated as a predictive biomarker of response to RTK inhibition in future MPNST clinical trials.

Even though p53-deficiency promoted resistance to MET and MEK inhibition, we found that p53 loss actually increased sensitivity to mTOR inhibition. Combined MEK and mTOR inhibition is currently being evaluated in MPNST in a phase 2 clinical trial (NCT03433183) [65]. Early reports indicate only 2/21 enrolled patients responded to treatment [66]. It is unknown what genetic factors may have contributed to response in these patients. In genetically engineered mouse models of MPNST mTOR [67] or combined mTOR and MEK inhibition [68] were highly effective in a *Nf1/Tp53* inactivated model but relatively less effective in an p53 intact Δ*Nf1/* Δ*Pten* model [35]. Previously, *TP53* loss or mutation has been reported to either decrease [69, 70] or increase [59, 71, 72] sensitivity to mTOR inhibition, depending on the context. Similar to our findings, human rhabdomyosarcoma cells lacking functional p53 are sensitive to rapamycin, while overexpression of p53 induces rapamycin resistance [59]. In these studies mechanistically, in the absence of p53, rapamycin induced sustained activation of the ASK1/JNK signaling cascade resulting in persistent c-Jun hyperphosphorylation and subsequent p53-independent apoptosis [60]. Restoration of p53 expression resulted in rapamycin resistance by inducing cell cycle arrest instead of JNK/c-Jun-mediated apoptosis [59, 60]; however, we found no evidence of substantial apoptosis in the context of mTOR inhibition in MPNST. Rather, mTOR and MEK inhibition induced Schwann cell and neuronal differentiation exclusively in p53 null cells in association with increased treatment sensitivity.

Induction of differentiation using epigenetic therapies has been proposed as a treatment for sarcomas [73], however our data indicate that kinase inhibitors may also be used to induce differentiation and cell cycle arrest in MPNSTs. Additionally, we show p53 loss plays a paradoxical role in differentiation therapy response, by causing resistance to MET inhibition and increased sensitivity to mTOR inhibition. Normal Schwann cells are capable of undergoing dedifferentiation followed by redifferentiation in cases of peripheral nerve injury to support nerve repair [74]. However, these reprogramming pathways are altered in disease states, including NF1, resulting in induction of bone, cartilage, muscle, and adipocyte differentiation pathways [75, 76]. p53 is also a critical regulator of reprogramming, as well as tissue development and mesenchymal stem cell differentiation pathways [77, 78]. This less conventional role of p53 may be particularly important for sarcomas such as MPNST, as additional loss of p53 in the context of NF1-defficiency may further contribute to deregulation of these key differentiation/reprogramming pathways to cause lineage plasticity. As an added layer of complexity, both MET and mTOR also regulate these processes. Upon nerve injury, MET [79] and mTOR [80] are required for the reprogramming of mature Schwann cells into dedifferentiated repair Schwann cells. In addition to this, MET plays a key role in embryonic muscle and nervous system development [81], while mTOR regulates bone development and chondrocyte differentiation [82, 83]. It remains unclear how differential lineage plasticity so precisely directs drug response in the presence or absence of p53 in MPNST. Differentiation therapy has been proposed as a p53-independent therapeutic strategy for p53-mutant carcinomas [84], but our data suggests that in MPNSTs there are also alternative p53-dependent mechanisms guiding differentiation therapy response. Collectively, these data suggest that there are key tissue-specific functions of p53 that contribute to drug response in MPNST and other sarcomas outside of the more classical tumor suppressor functions of p53.

## Conclusion

Our data reveals profound kinome signaling plasticity in MPNST cells and a complex interplay between clonal subpopulations that is influenced by p53. Apart from the well-known tumor suppressor role of p53, we demonstrate that p53-deficiency promotes acquired resistance to targeted kinase inhibition by modulating kinome signaling and MET localization. Moreover, p53-deficiency enhances lineage plasticity which also contributes to kinase inhibitor resistance. Understanding how p53 and other commonly altered genes modulate treatment response is critical for the advancement of precision medicine approaches for MPNST patients.

## Methods

### Cell culture and drugs

Mouse-derived MPNST cell lines described previously [34] were cultured at 37 °C in a humidified atmosphere in 5% CO_2_ in low pH DMEM (Thermo Fisher Scientific) supplemented with 10% FBS (Corning, lot #35070165) and 1% Penicillin Streptomycin (Thermo Fisher Scientific), unless otherwise indicated. Cell lines were verified to be free of *Mycoplasma* contamination every 6 months by PCR (ATCC). Human-derived MPNST cell lines were cultured as described previously [85]. Capmatinib (Novartis), trametinib (Novartis), everolimus (Selleckchem), and afuresertib (Selleckchem) solutions were prepared in DMSO. For human cell line [86] IC_50_ experiments, drugs were purchased as 10 mM stock solutions (Selleckchem) and handled as previously described [85]. All cell culture experiments were performed 3 independent times, unless otherwise noted.

### Viability and dose response

For cell viability experiments, cells were allowed to adhere and then treated with DMSO or the indicated drug dose. After 72 h, cells were trypsinized (Thermo Fisher Scientific) and trypan blue negative cells were counted using a TC20 Automated Cell Counter (Bio-Rad) to determine percent viability relative to vehicle treatment. To meet the assumption of equal variance, data were log transformed and pairwise comparisons of beta regressions were done using the betareg (v 3.1-3) and emmeans (v 1.4.5) packages and plotted using the ggplot2 (v 3.3.0) package in R (v 3.6.3). Emmeans and ggplot2 were also used for comparison of doubling times. For human MPNST cell lines, the IC_50_ was determined as previously described [85]. Briefly, cells were allowed to adhere and then treated with DMSO or the indicated serially diluted drug (4.6 nM–10 μM) for 72 h, cell viability was measured by ATPlite Luminescence Assay (PerkinElmer), and IC_50_ was calculated using GraphPad Prism 7. The rcorr function in the Hmisc (v4.4-1) package was used to generate spearman correlations of IC_50_. The ggplot2 (v 3.3.0) and ggpubr (v 0.2.5) packages were used to plot an IC_50_ matrix and correlogram, respectively, using R (v 3.6.3). For dose combination matrices, 2500 cells/well were plated in a 96 well plate, allowed to adhere, and then treated with the indicated serially diluted drug or vehicle for 72 h. Cell viability was measured using CellTiter 96 Aqueous One Solution Cell Proliferation Assay (MTS) (Promega) following the manufacture’s protocol. Absorbance was read using Synergy Neo microplate reader (BioTek) and normalized to cell number using a standard curve generated by GraphPad Prism 7. Mean percent inhibitions relative to vehicle controls were plotted in dose response matrices using the synergyfinder package (v 2.0.12) in R (v 3.6.3).

### Reverse phase protein array

For cell culture experiments, cells were seeded in a 6-well plate, allowed to adhere overnight, and then treated in replicates of 6 with DMSO or the indicated drug or ligand dose for 2 or 48 h. To harvest samples, the cells were washed thoroughly 3 times with ice cold PBS, and plates were immediately snap frozen on dry ice to preserve the integrity of the phosphoproteome. For mouse tumorgrafts, we analyzed data, where some of this data was used in a previous study [25]. Briefly, immediately following the euthanasia of tumor-bearing mice, 15–25 mg portions of each tumor were transplanted into the flank of NSG-SCID mice using a 10-gauge trochar. When the tumor volume reached approximately 150 mm^3^, mice were euthanized, and tumors were immediately harvested and snap-frozen in liquid nitrogen within 20 min upon surgical resection to preserve the integrity of the phosphoproteome. Six tumors were assessed for each genotype. All animal experimentation in this study was approved by the Van Andel Institute’s Internal Animal Care and Use Committee (XPA-19-04-001). Specimens were then embedded in an optimal cutting temperature compound (Sakura Finetek, Torrance, CA, USA), cut into 8 μm cryo-sections, mounted on uncharged glass slides, and stored at −80 °C until use. Each slide was fixed in 70% ethanol (Sigma Aldrich, Darmstadt, Germany), washed in deionized water, stained with hematoxylin (Sigma Aldrich, Darmstadt, Germany) and blued in Scott’s Tap Water substitute (Electron Microscopy Sciences), and dehydrated through an ethanol gradient (70%, 95%, and 100%) and xylene (Sigma Aldrich, Darmstadt, Germany). In order to prevent protein degradation, complete protease inhibitor cocktail tablets (Roche Applied Science, Basel, Switzerland) were added to the ethanol, water, hematoxylin, and Scott’s Tap Water substitute [87]. Cells were lysed in a 1:1 solution of 2× Tris-Glycine SDS Sample buffer (Invitrogen Life Technologies, Carlsbad, CA, USA) and Tissue Protein Extraction Reagent (Pierce, Waltham, MA, USA) supplemented with 2.5% of 2-mercaptoethanol (Sigma Aldrich, Darmstadt, Germany). Cell lysates were boiled for 8 min and stored at −80 °C. Reverse phase protein microarray construction and immunostaining was performed as previously described [25] A total of 98 protein sites passed quality control metrics and were used for analysis. Differential expression was performed using R package “limma” [88] and R (v 3.6.0). For differential activation analysis, *P*-values for delta-delta differences in limma fold changes were generated in R (v 3.6.3) using Wald tests and adjusted using the BH method. Adjusted *P*-values of less than 0.05 were considered significant. For tumors, the pairwise difference between genotypes in mean centered phospho-site expression (Z score) was assessed using a linear mixed-effects model with a random intercept for each phospho-site using the lme4 (v 1.1-23) and emmeans (v 1.4.5) packages in R with the selected phospho-sites. Pairwise differences between genotypes in log transformed expression were also assessed for each individual phospho-site using emmeans. A comprehensive analysis including all 98 tumorgraft phospho-sites was previously published [25] Balloon plots were created from the limma fold changes (cell lines) or z-scores (tumors) in R (v 3.6.3) using the ggballoonplot function in the ggpubr package (v 0.2.5). Labeled fold change, differential activation, waterfall, and site-specific expression plots were created using ggplot2 (v 3.3.0) in R (v 3.6.3).

### Lentiviral transduction

To generate the NF1-MET;sgP53 cell line, the pLentiCRISPRv2 vector (Addgene #52961) [89] was digested with BsmBI and dephosphorylated with CIP (New England BioLabs) following the manufactures instructions and then purified with using Qiaquick Gel Extraction kit (Qiagen). The oligonucleotides 5’-cac cgA GCC AAG TCT GTT ATG TGC A-3’ and 5’-aaa cTG CAC ATA ACA GAC TTG GCT c-3’ were annealed and then ligated into the vector using T4 PNK and T4 ligase (Thermo Fisher Scientific). One Shot competent cells (Thermo Fisher Scientific) were transformed and candidate colonies were verified by diagnostic digestion. Lentivirus was produced by lipofectamine transfection (Thermo Fisher Scientific) of HLA 293 cells kindly provided by Dr. Bart Williams with either the pLentiCRISPRv2;sgP53 plasmid or the pLentiCRISPRv2 plasmid (as an empty vector control), along with the envelop and packaging plasmids pCMV-VSV-G (Addgene #8454) [90] and psPAX2 (Addgene #12260). NF1-MET cells were infected with 500 μL of 0.45 μm filtered virus with polybrene (EMD Millipore), and 48 h post infection transduced cells were selected for by 2 μg/mL puromycin (Thermo Fisher Scientific) treatment. To generate H2B GFP and RFP labeled cells, lentivirus was produced by lipofectamine transfection of Phoenix 293 cells kindly provided by Dr. Bart Williams with either LV-GFP (Addgene #25999) [91] or pHIV-H2BmRFP (Addgene #18982) [92] plasmids, along with pCMV-VSV-G and psPAX2. Filtered lentivirus was concentrated using Lenti-X Concentrator (Takara). NF1-MET;sgEmptyVector and NF1-MET;sgP53 cells were infected with GFP or RFP lentivirus to create NF1-MET-GFP and NF1-MET;sgP53-RFP cell lines, respectively.

### Competition assay

For clonal competition assays, single cell suspensions of NF1-MET-GFP and NF1-MET;sgP53-RFP cells were prepared in sorting buffer (HBSS without Ca^2+^, Mg^2+^, and Phenol Red (Thermo Fisher Scientific) with 25 mM HEPES, 2 mM EDTA, 2% FBS, and 10 mg/mL DAPI added). 7,500 GFP+ and 7,500 RFP+ single live cells were sorted into each well of a 24 well culture plate containing low pH DMEM with 10% FBS using a MoFlo Astrios cell sorter with Summit v6.3 software (Beckman Coulter). Sorting was performed at 25 psi using a 100um nozzle, with Purify for the abort mode and a drop envelope set to 1–2 drops. Cells were selected using SSC vs FSC, and single cells using both SSC area vs height and area vs width. Live cells (DAPI negative) were identified using the 355-448/59 channel, and GFP and RFP signals were detected using the 488-510/20 and 561-614/20 channels, respectively.

After cells adhered they were treated with DMSO or the indicated drug dose and cocultured for the indicated timepoints. The cocultured cells were analyzed to determine the percentage of single, live cells that were either GFP+ or RFP+ using a CytoFLEX S flow cytometer with CytExpert v2.4 software (Beckman Coulter). Analysis was performed using FlowJo v10.7 software (BD Life Sciences). Single cells were identified using SSC vs FSC, followed by SSC area vs height. Live cells (DAPI negative) were identified using the 405-450/45 channel, and GFP and RFP signals were detected using the 488-525/40 and 561-610/20 channels, respectively. Representative images of the gating strategy are shown in Supplementary Fig. 1. Pairwise comparisons of beta regressions were done using the betareg (v 3.1-3) and emmeans (v 1.4.5) packages and plotted using the ggplot2 (v 3.3.0) package in R (v 3.6.3).

### Western blotting

Cells were grown overnight followed by serum starving overnight for a final confluency of 90%, and then treated with drug and/or stimulated with 10% serum or 100 ng/mL HGF or EGF as indicated. Cell lysate collection and immunoblotting were done as previously described [34]. Primary antibodies were purchased from Cell Signaling: p53 (#2524), phospho-MET Y1234/1235 (#3077), MET (#3127), phospho-AKT S473 (#9271), phospho-ERK T202/Y204 (#9101), phospho-S6 S235/236 (#4858), β-Actin (#3700).

### RT-qPCR

RNA was isolated using RNeasy Mini Kit and RNase-Free DNase set (Qiagen) following the manufactures protocol. cDNA was synthesized using SuperScript II Reverse Transcriptase with Oligo (dT) 12-18mer Primers (Thermo Fisher Scientific) using Tetrad 2 (BioRad) following the manufactures protocols. qPCR was done on Step One Plus (Applied Biosystems) using Fast Start Universal SYBR Green Master Mix (Roche). Primer sequences are listed in Table 2. Relative expression was calculated using the ddCt method and plotted using GraphPad Prism 7 (*Cdkn1a*) or using the ggplot2 (v 3.3.0) package in R (v 3.6.3). For statistical analysis, a linear mixed-effects model with random intercepts for experimental and technical replicates was used to summarize relative expression from 4 separate experiments using the lme4 (v 1.1-23) and emmeans (v 1.4.5) packages in R. All p-values were automatically adjusted using the Tukey method.

**Table 2:** Primer sequences.

Gene	Forward	Reverse
Acta2	CGAAACCACCTATAACAGCATCA	GCGTTCTGGAGGGGCAAT
Cdkn1a	CTTGCACTCTGGTGTCTG	CTTGGAGTGATAGAAATCTGTCA
Cebpa	AGGTGCTGGAGTTGACCAGT	CAGCCTAGAGATCCAGCGAC
Col11a1	GACCAGAAGACACACTGAAAGCA	TCCATGCCATCTGAGTAGTCAAGA
Fabp7	CTCTGGGCGTGGGCTTT	TTCCTGACTGATAATCACAGTTGGTT
Fbln2	AGTGGCCGTAAGTATGCTGC	GGAAGCTGGTAGCAAATGAGC
Il1B	TGTAATGAAAGACGGCACACC	TCTTCTTTGGGTATTGCTTGG
Il10	CCCTTTGCTATGGTGTCCTT	TGGTTTCTCTTCCCAAGACC
Il6	GAGGATACCACTCCCAACAGACC	AAGTGCATCATCGTTGTTCATACA
Map2	TCTAAAGAACATCCGTCACAGG	GGTGAGCATTGTCAAGTGAGC
Mcp1	GCATCCACGTGTTGGCTCA	CTCCAGCCTACTCATTGGGATCA
Ppia	GGCAAATGCTGGACCAAAC	CATTCCTGGACCCAAAACG
S100B	CTGGAGAAGGCCATGGTTGC	CTCCAGGAAGTGAGAGAGCT
Sox9	TCCACGAAGGGTCTCTTCTC	AGGAAGCTGGCAGACCAGTA
Spp1	TCTCCTTGCGCCACAGAATG	TCCTTAGACTCACCGCTCTT
Synpo	CATCGGACCTTCTTCCTGTG	TCGGAGTCTGTGGGTGAG
Tubb5	ATGCCATGTTCATCGCTTAT	TTGTTCGGTACCTACATTGG
Vcam1	TCGCTCAAATCGGTGACTC	ACAGGCTCCATGGTCAGAAC

### Mouse xenograft models

Six- to eight-week-old female NSG-SCID mice were injected subcutaneously with one million cells of either NF1-MET or NF1-MET;sgP53 (10 mice/cell line). Mice were randomly assigned to each treatment group by tumor growth rate, so that growth rates were matched across treatment groups. The investigator was not blinded. Tumors were measured twice weekly with calipers. Once tumors reached approximately 150 mm^3^, 5 mice from each cell line group were treated with either 30 mg/kg capmatinib or vehicle (0.5% methylcellulose) twice daily for 15 days or until tumors reach 2500 mm^3^. A second experiment was performed exactly the same way, for a final total of 10 mice in each cell line and treatment group. All animal experimentation in this study was approved by the Van Andel Institute’s Internal Animal Care and Use Committee (XPA-19-04-001). Sample sizes were selected based on our previously published study of NF1-MET tumorgrafts [34].

A linear mixed-effects model was used to determine if there were significant differences between the results of experiment one and experiment two. The models were stratified by treatment group. Data were square root transformed before being entered into the model. The model used a 3-way interaction for timepoint, cell-line, and experiment and also included a random intercept for tumor ID. A linear mixed-effects model was used for the combination analysis. Data were square root transformed before being entered into the model. This was a linear mixed effects model with a 4-way interaction between timepoint, experiment, treatment group, and cell-line. A random intercept for tumor-id was also included. All statistical analyses were run using R (v. 3.6.0) and all linear regression models were run using the lme4 package. The emtreands function from the emmeans package was used for the contrasts/comparisons that were run on the regression output to obtain growth trend estimates for the average tumor growth rate for each treatment/genotype group. All p-values that were obtained through the emmeans output were automatically multiple-testing corrected via the Tukey method.

### Immunofluorescence

Cells were plated at 50,000 cells per well on glass coverslips (Fisherbrand) in a 24 well plate and allowed to attach overnight. The next day cells were serum-starved for 16 h. The third day cells were treated with 100 ng/mL HGF for 5 min at 37 °C, washed three times with phosphate buffered saline + 0.5% tween 20 (PBST), and fixed with 4% formaldehyde in PBS for 15 min. Cells were permeabilized with ice-cold methanol for 3 min at −20 °C. Samples were blocked (5% normal goat serum, 1% BSA, 0.3% Triton X-100 in PBS) for 1 h at room temperature in a humidity chamber. Antibodies against phospho-MET (Y1230/1234/1235; Abcam #5662) were diluted 1:100 in antibody dilution buffer (1% BSA and 0.3% triton-x 100 in PBS) and incubated with the cells overnight at 4 °C in a humidity chamber. Primary antibodies were detected using Alexa Flour secondary antibody goat-against-rabbit 594 (Life Technologies #8889) at 1:500 and incubated for 40 min at room temperature. Cell nuclei were stained with DAPI and samples were mounted with ProLong Gold antifade reagent (Life Technologies). For imaging, at three regions of interest were imaged per sample using a 60x Plan Apo VC oil immersion objective with 1.4 NA on a Nikon A1 plus-RSi laser scanning confocal microscope (Nikon Elements software). Image resolution was 1024 × 1024 of z-slices covering the entirety of cell thickness. PMT levels were set using controls with 403 and 561 solid-state lasers. FIJI was used to generate maximum intensity projection TIFF images.

### RNA sequencing

Cells were plated in a 6-well plate, grown in 10% FBS overnight, and then treated in duplicate with 100 nM capmatinib or DMSO. RNA was isolated using RNeasy Mini Kit and RNase-Free DNase set (Qiagen) following the manufactures protocol. Libraries were prepared by the Van Andel Genomics Core from 500 ng of total RNA using the KAPA mRNA Hyperprep kit (v4.17) (Kapa Biosystems, Wilmington, MA USA). RNA was sheared to 300–400 bp. Prior to PCR amplification, cDNA fragments were ligated to IDT for Illumina TruSeq UD Indexed adapters (Illumina Inc, San Diego CA, USA). Quality and quantity of the finished libraries were assessed using a combination of Agilent DNA High Sensitivity chip (Agilent Technologies, Inc.), QuantiFluor® dsDNA System (Promega Corp., Madison, WI, USA), and Kapa Illumina Library Quantification qPCR assays (Kapa Biosystems). Individually indexed libraries were pooled and 50 bp, paired end sequencing was performed on an Illumina NovaSeq6000 sequencer using an S2, 100 bp sequencing kit (Illumina Inc., San Diego, CA, USA) to a minimum raw depth of 41.4 M reads with an average of 47.2 M reads per sample. Base calling was done by Illumina RTA3 and output of NCS was demultiplexed and converted to FastQ format with Illumina Bcl2fastq v1.9.0. Sequencing adapters were trimmed using Trimgalore v0.4.2 (http://www.bioinformatics.babraham.ac.uk/projects/trim_galore/). Bases with a quality score less than 20 were also removed from the ends of reads. Trimmed data were quality controlled with FastQC v0.11.7 [93] and then mapped with STAR v2.5.2b to the mm10 genome using the default settings [94]. Raw gene counts (minimum of 30 M counts per sample and a mean of 34.92) generated by STAR were imported into R v3.6.0. Genes with less than 10 counts in a minimum of two samples were immediately removed from all further analysis; this minimizes multiple testing adjustments and removes low-expressed genes unlikely to be biologically meaningful. For all differential expression contrasts using a subset of the data, the count data were further filtered so that, for the samples being used in the contrast, a minimum of two samples have > 0 counts. A quasi-likelihood negative binomial generalized log-linear model was then fit to the filtered count data using the weighted trimmed mean of M-values to normalize for library size and composition biases [95]. *P*-values were generated using empirical Bayes quasi-likelihood F-tests, and then adjusted using the BH method; adjusted *P*-values less than 0.05 were considered significant. Gene ontology enrichment analyses were done using the clusterProfiler R package v3.14.3 using the function ‘goseq’ with KEGG ontologies. Heatmaps were generated from library-size normalized counts centered across genes (z-scores) using the pheatmap package v1.0.12.

### Apoptosis assay

NF1-MET and NF1-MET;sgP53 cells were plated in a 6-well plate, grown in 10% FBS overnight, and then treated with 20 nM everolimus and 40 nM trametinib or DMSO for the indicated time points. Cells were harvested and stained with Annexin V Alexa Fluor 647 Conjugate (Invitrogen) and DAPI according to the manufactures protocol. Stained samples were acquired on a Beckman Coulter CytoFLEX S running CytExpert v2.4. AnnexinV-A647 and DAPI were detected in the 640-660/20 and 405-450/45 channels respectively. Data were analyzed using FlowJo v10.x. An initial gate was set on a forward vs side scatter plot to include both live and dead cells while excluding small debris and noise. Aggregated cells were removed for analysis using a gate on side scatter area vs side scatter height. The single cells were then analyzed for health on a plot of Annexin V vs DAPI. For statistical analysis, the Annexin V and/or DAPI positive cell percentages from 4 separate experiments were calculated using a beta family generalized linear mixed effect model with a random intercept for the experimental run and a logit link using glmmTMB (v1.1.3) in R. Treatment verses control odds ratios were assessed using emmeans (v1.4.5).

### Supplementary information

Supplemental Figure Legends

Supplemental Figures

## Data Availability

The RNA-seq data generated in this study were deposited in NCBI’s Gene Expression Omnibus and are accessible through GEO Series accession number GSE225747 (https://www.ncbi.nlm.nih.gov/geo/query/acc.cgi?acc=GSE225747).

## Code availability

R code used in this study is available upon request.
