# Supplementary material for: p53 modulates kinase inhibitor resistance and lineage plasticity in NF1-related MPNSTs
Source: Oncogene. 2024 Mar 13;43(19):1411–30. doi: 10.1038/s41388-024-03000-9 (PMC11068581; doi:10.1038/s41388-024-03000-9)
Supplement: Supplementary file 2 — Supplemental Figures [file 41388_2024_3000_MOESM2_ESM.pptx]

## Slide 1
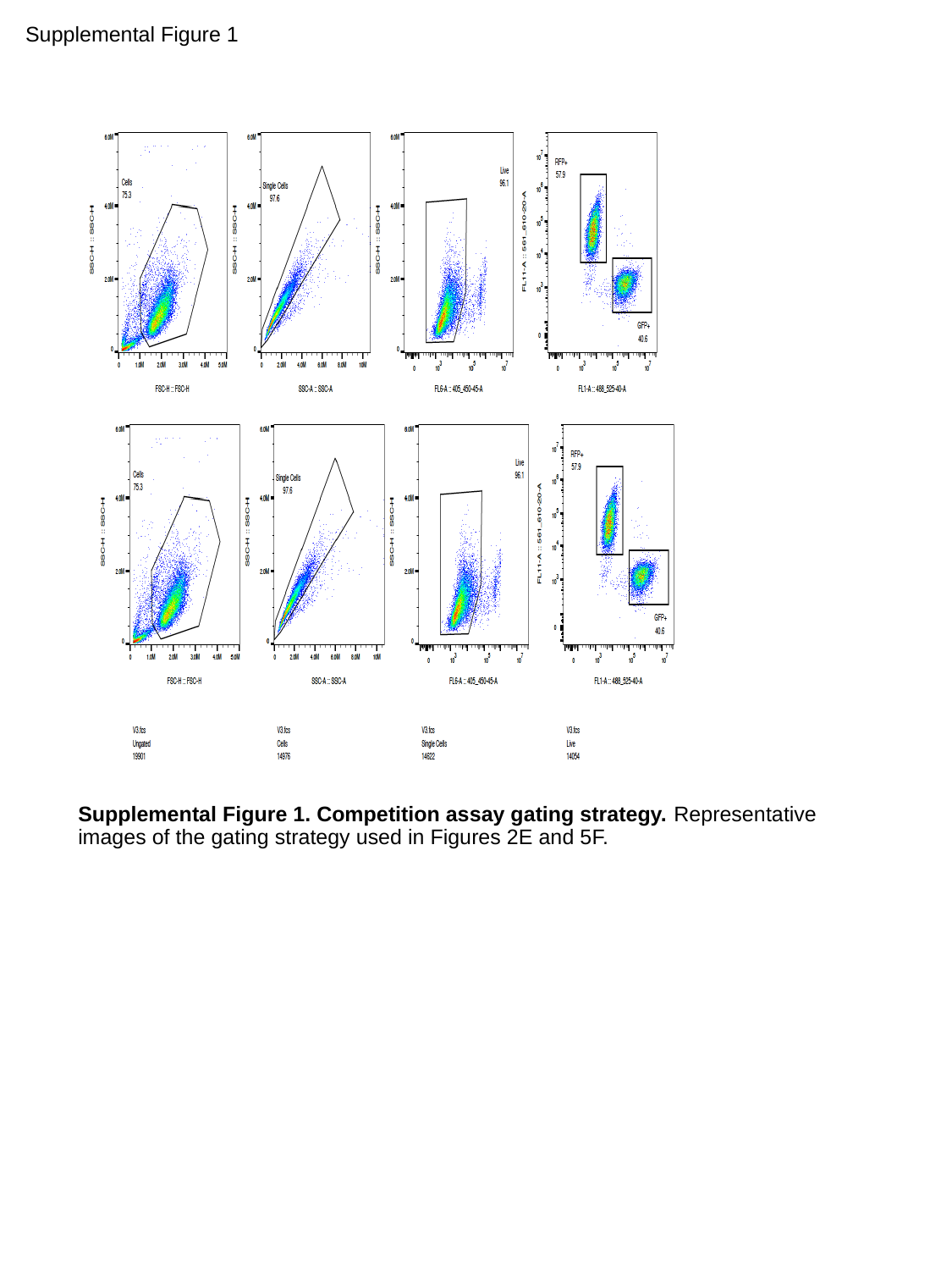

Supplemental Figure 1
Supplemental Figure 1. Competition assay gating strategy. Representative images of the gating strategy used in Figures 2E and 5F.

## Slide 2
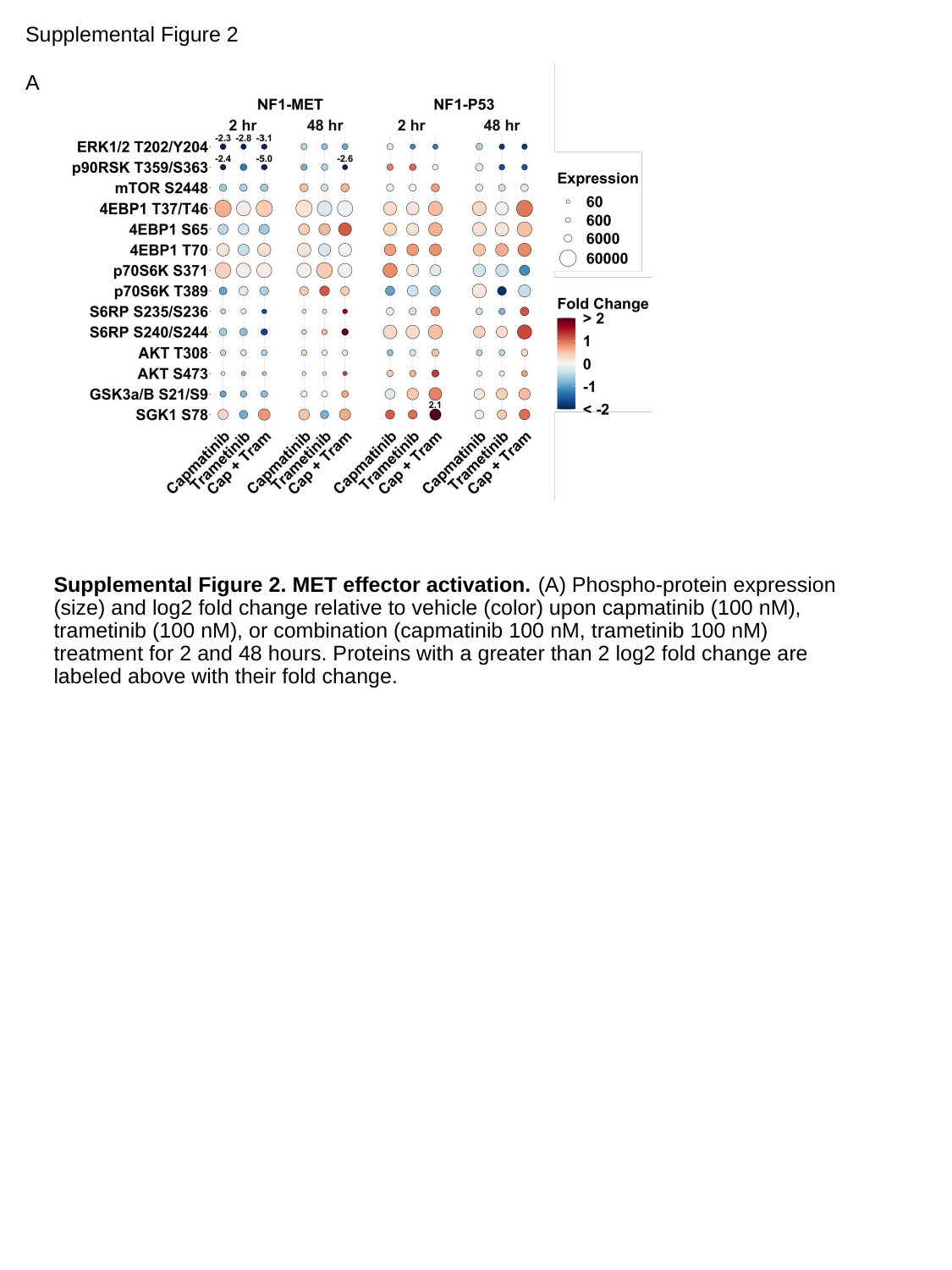

Supplemental Figure 2
A
Supplemental Figure 2. MET effector activation. (A) Phospho-protein expression (size) and log2 fold change relative to vehicle (color) upon capmatinib (100 nM), trametinib (100 nM), or combination (capmatinib 100 nM, trametinib 100 nM) treatment for 2 and 48 hours. Proteins with a greater than 2 log2 fold change are labeled above with their fold change.

## Slide 3
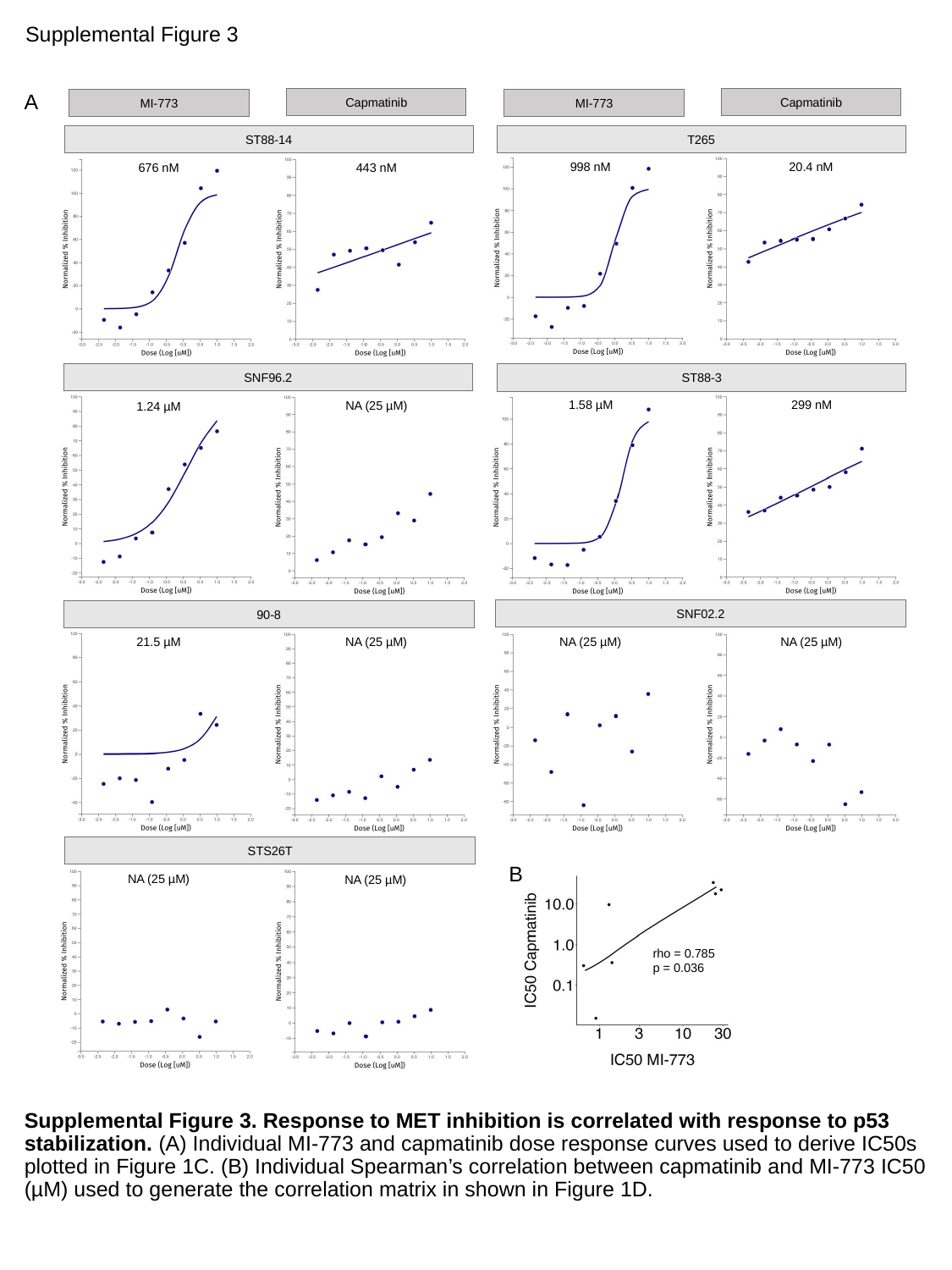

Supplemental Figure 3
A
Capmatinib
Capmatinib
MI-773
MI-773
T265
ST88-14
998 nM
20.4 nM
443 nM
676 nM
SNF96.2
ST88-3
1.58 µM
299 nM
NA (25 µM)
1.24 µM
SNF02.2
90-8
NA (25 µM)
NA (25 µM)
NA (25 µM)
21.5 µM
STS26T
B
NA (25 µM)
NA (25 µM)
rho = 0.785
p = 0.036
Supplemental Figure 3. Response to MET inhibition is correlated with response to p53 stabilization. (A) Individual MI-773 and capmatinib dose response curves used to derive IC50s plotted in Figure 1C. (B) Individual Spearman’s correlation between capmatinib and MI-773 IC50 (µM) used to generate the correlation matrix in shown in Figure 1D.

## Slide 4
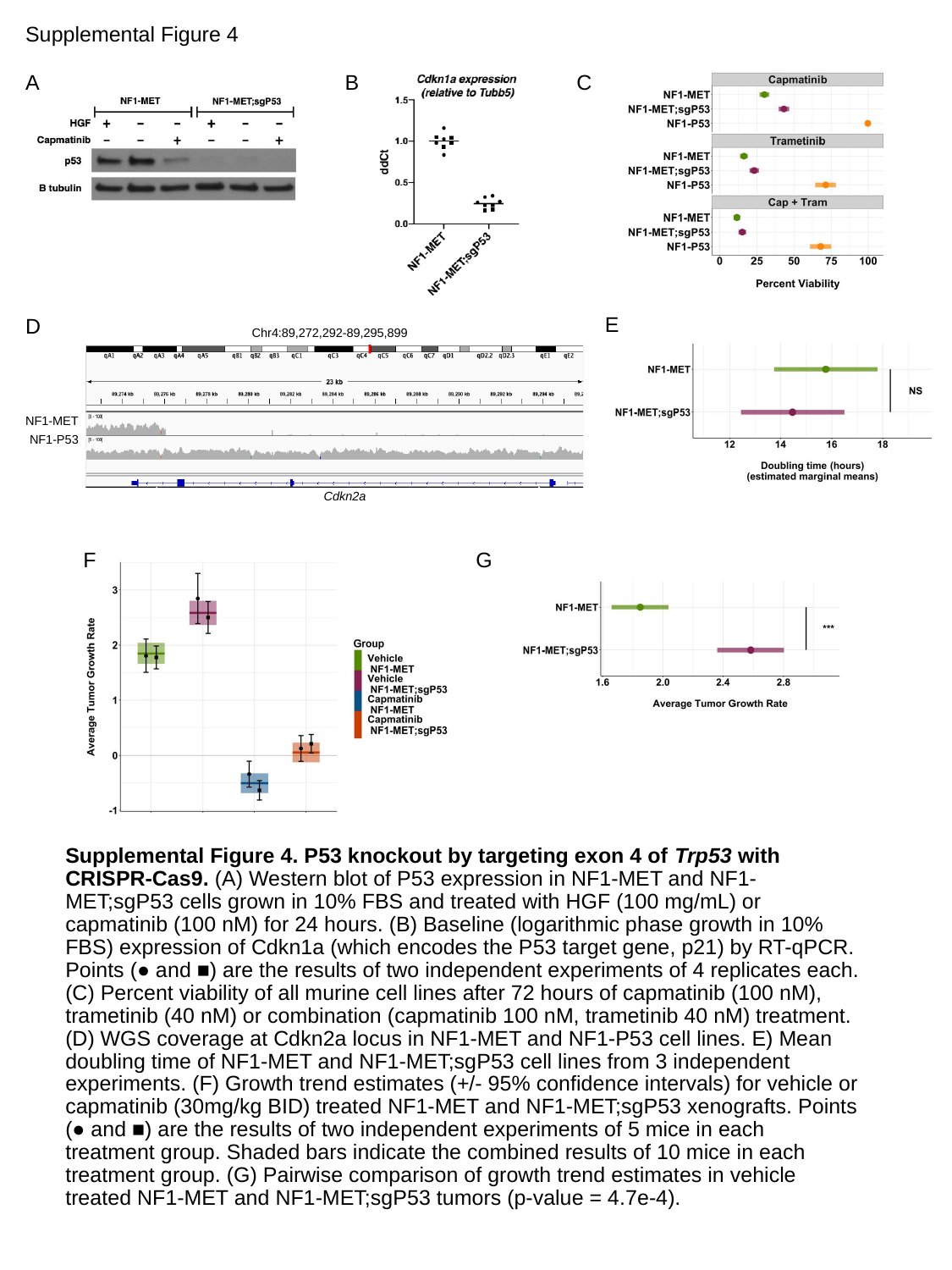

Supplemental Figure 4
C
A
B
E
D
Chr4:89,272,292-89,295,899
NF1-MET
NF1-P53
Cdkn2a
G
F
Supplemental Figure 4. P53 knockout by targeting exon 4 of Trp53 with CRISPR-Cas9. (A) Western blot of P53 expression in NF1-MET and NF1-MET;sgP53 cells grown in 10% FBS and treated with HGF (100 mg/mL) or capmatinib (100 nM) for 24 hours. (B) Baseline (logarithmic phase growth in 10% FBS) expression of Cdkn1a (which encodes the P53 target gene, p21) by RT-qPCR. Points (● and ■) are the results of two independent experiments of 4 replicates each. (C) Percent viability of all murine cell lines after 72 hours of capmatinib (100 nM), trametinib (40 nM) or combination (capmatinib 100 nM, trametinib 40 nM) treatment. (D) WGS coverage at Cdkn2a locus in NF1-MET and NF1-P53 cell lines. E) Mean doubling time of NF1-MET and NF1-MET;sgP53 cell lines from 3 independent experiments. (F) Growth trend estimates (+/- 95% confidence intervals) for vehicle or capmatinib (30mg/kg BID) treated NF1-MET and NF1-MET;sgP53 xenografts. Points (● and ■) are the results of two independent experiments of 5 mice in each treatment group. Shaded bars indicate the combined results of 10 mice in each treatment group. (G) Pairwise comparison of growth trend estimates in vehicle treated NF1-MET and NF1-MET;sgP53 tumors (p-value = 4.7e-4).

## Slide 5
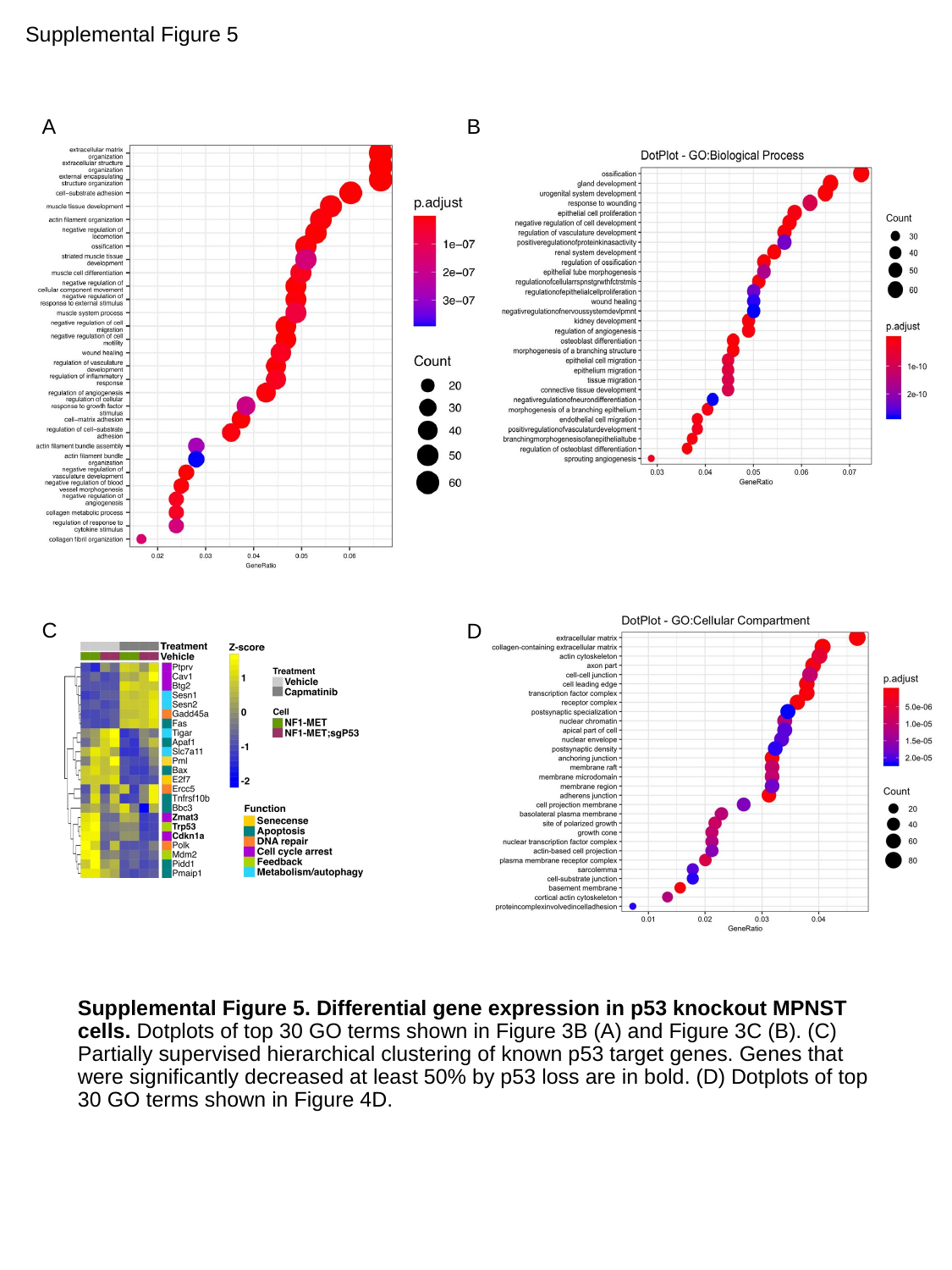

Supplemental Figure 5
A
B
C
D
Supplemental Figure 5. Differential gene expression in p53 knockout MPNST cells. Dotplots of top 30 GO terms shown in Figure 3B (A) and Figure 3C (B). (C) Partially supervised hierarchical clustering of known p53 target genes. Genes that were significantly decreased at least 50% by p53 loss are in bold. (D) Dotplots of top 30 GO terms shown in Figure 4D.

## Slide 6
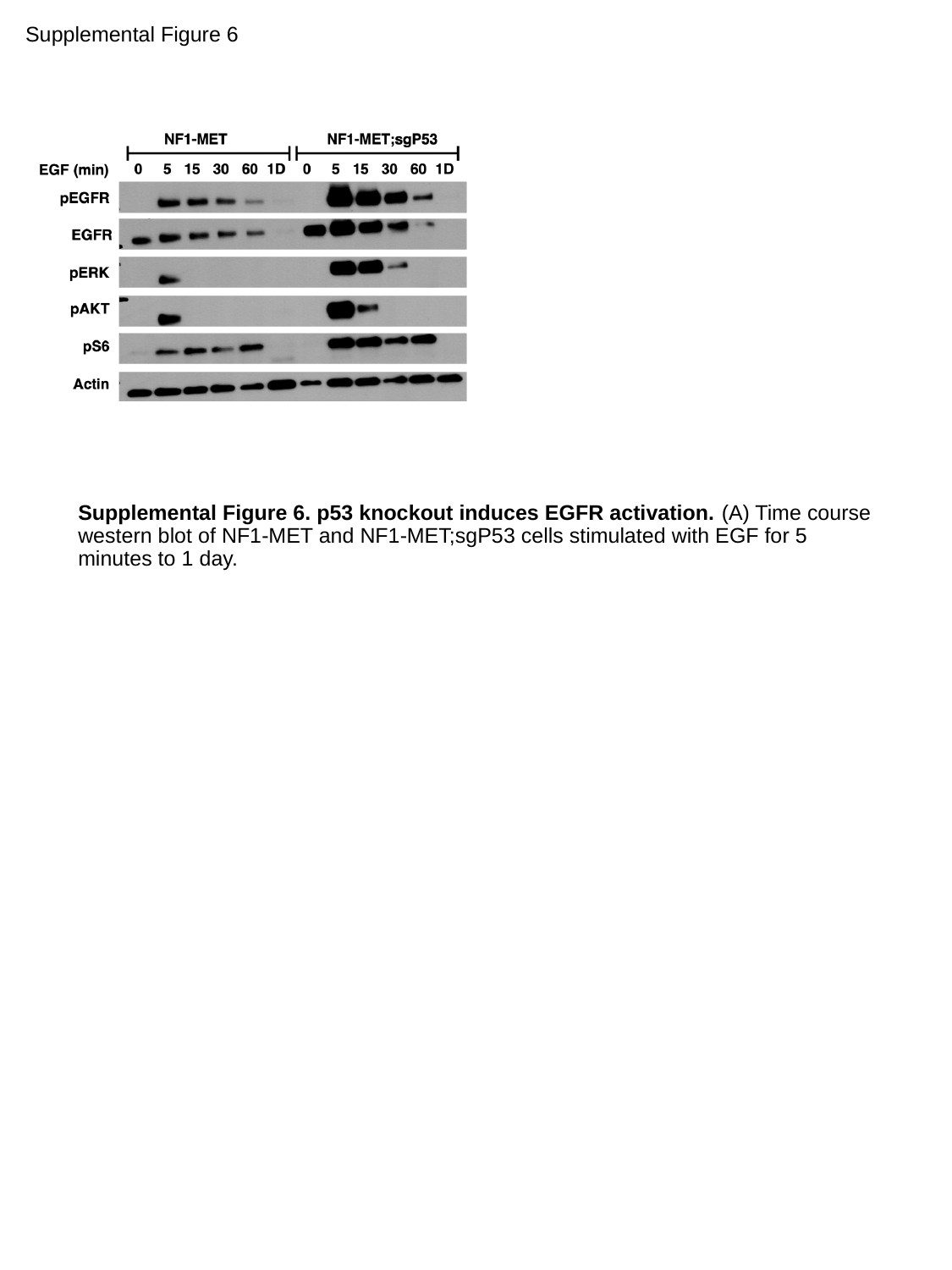

Supplemental Figure 6
Supplemental Figure 6. p53 knockout induces EGFR activation. (A) Time course western blot of NF1-MET and NF1-MET;sgP53 cells stimulated with EGF for 5 minutes to 1 day.

## Slide 7
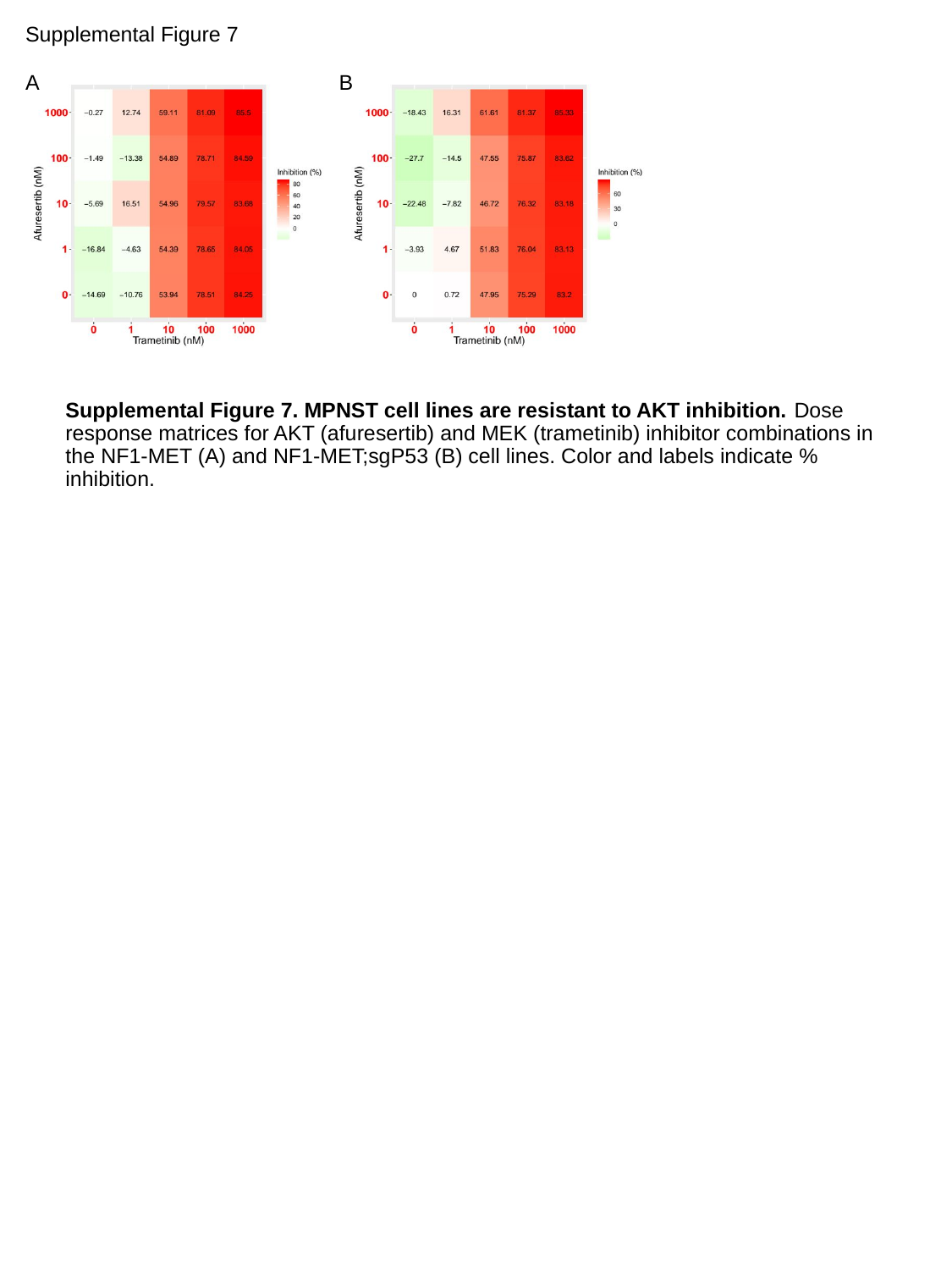

Supplemental Figure 7
A
B
Supplemental Figure 7. MPNST cell lines are resistant to AKT inhibition. Dose response matrices for AKT (afuresertib) and MEK (trametinib) inhibitor combinations in the NF1-MET (A) and NF1-MET;sgP53 (B) cell lines. Color and labels indicate % inhibition.

## Slide 8
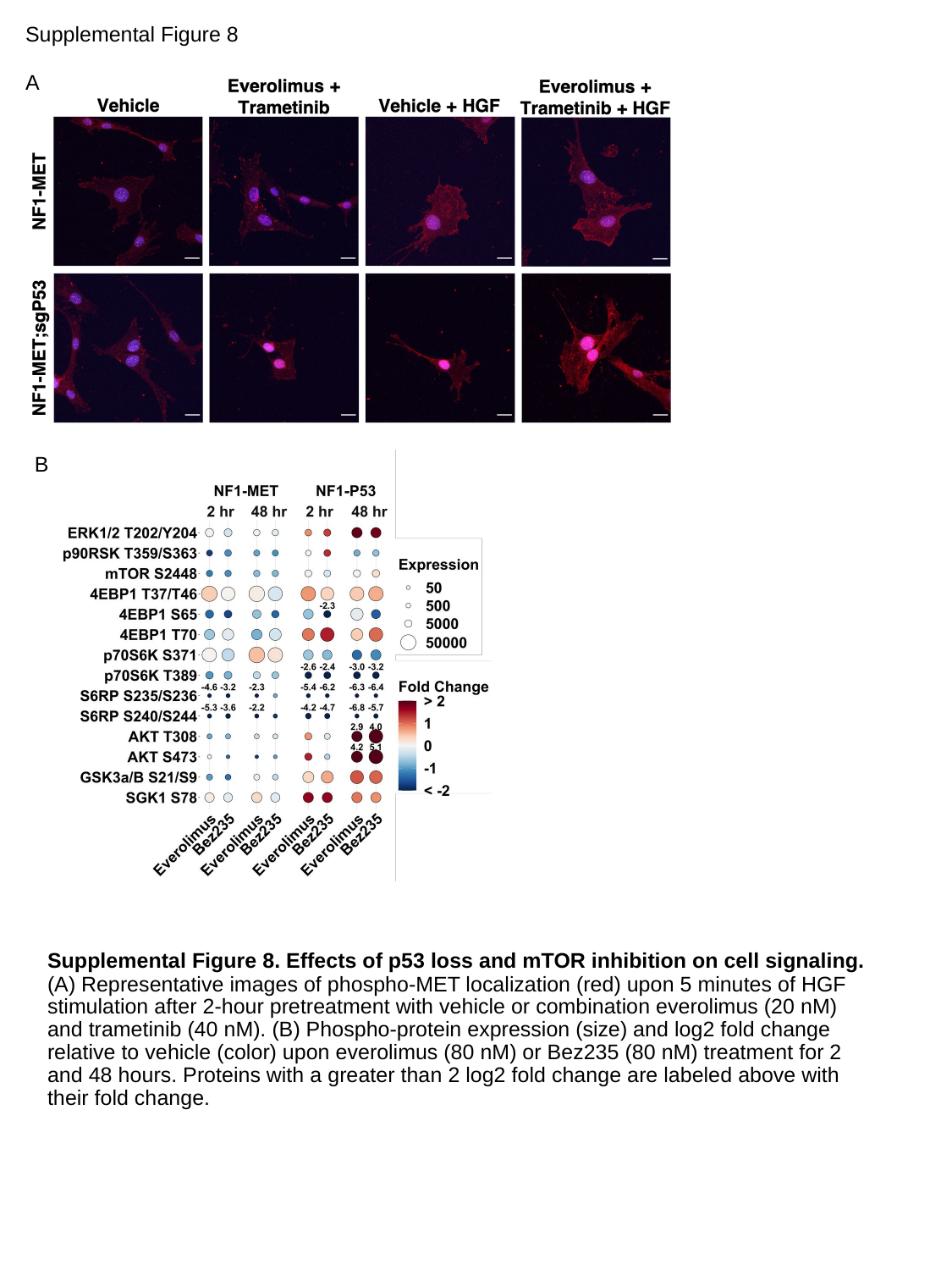

Supplemental Figure 8
A
B
Supplemental Figure 8. Effects of p53 loss and mTOR inhibition on cell signaling. (A) Representative images of phospho-MET localization (red) upon 5 minutes of HGF stimulation after 2-hour pretreatment with vehicle or combination everolimus (20 nM) and trametinib (40 nM). (B) Phospho-protein expression (size) and log2 fold change relative to vehicle (color) upon everolimus (80 nM) or Bez235 (80 nM) treatment for 2 and 48 hours. Proteins with a greater than 2 log2 fold change are labeled above with their fold change.

## Slide 9
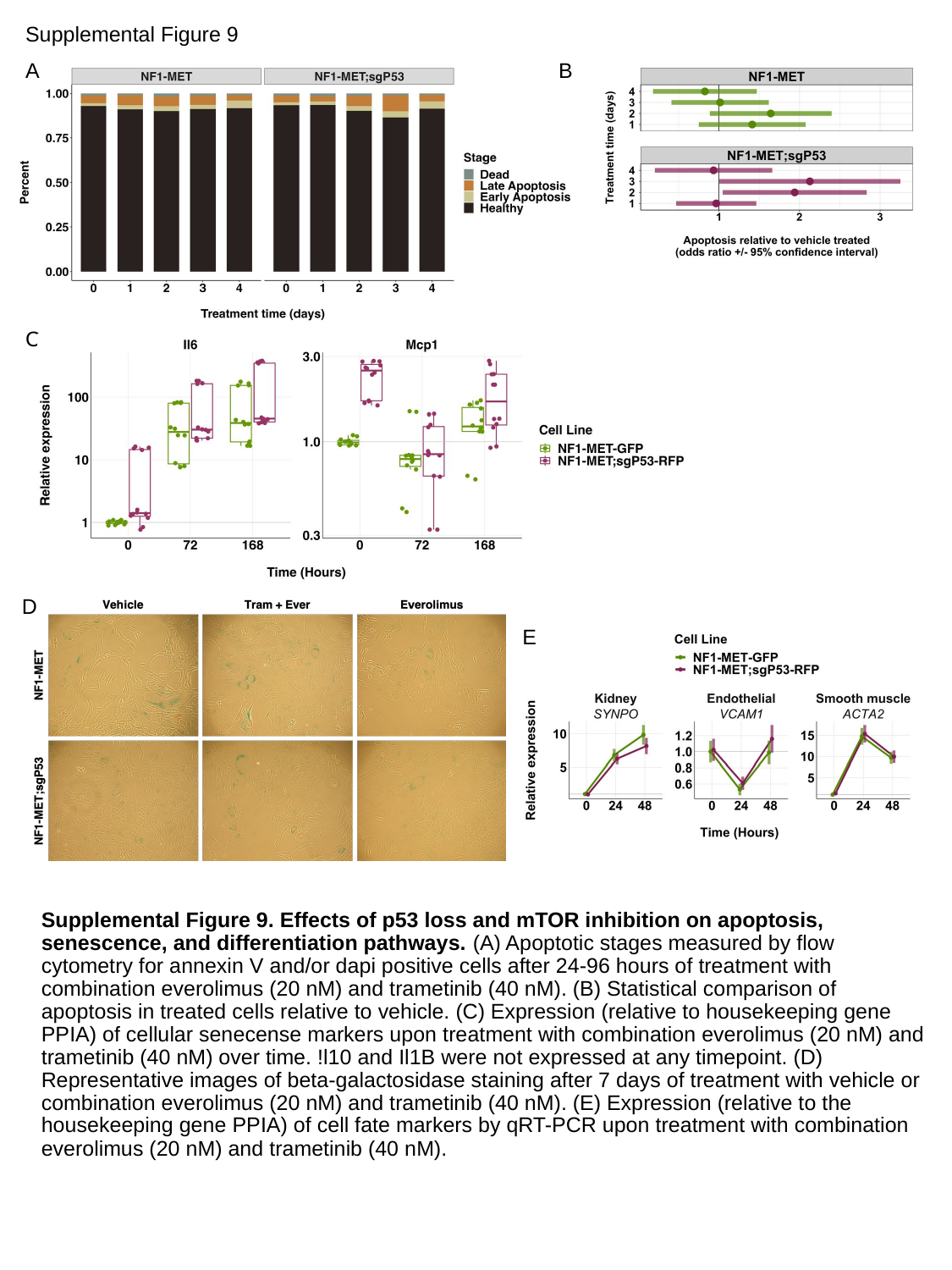

Supplemental Figure 9
B
A
C
D
E
Supplemental Figure 9. Effects of p53 loss and mTOR inhibition on apoptosis, senescence, and differentiation pathways. (A) Apoptotic stages measured by flow cytometry for annexin V and/or dapi positive cells after 24-96 hours of treatment with combination everolimus (20 nM) and trametinib (40 nM). (B) Statistical comparison of apoptosis in treated cells relative to vehicle. (C) Expression (relative to housekeeping gene PPIA) of cellular senecense markers upon treatment with combination everolimus (20 nM) and trametinib (40 nM) over time. !l10 and Il1B were not expressed at any timepoint. (D) Representative images of beta-galactosidase staining after 7 days of treatment with vehicle or combination everolimus (20 nM) and trametinib (40 nM). (E) Expression (relative to the housekeeping gene PPIA) of cell fate markers by qRT-PCR upon treatment with combination everolimus (20 nM) and trametinib (40 nM).
